# Analyses of dextroamphetamine and its metabolites in human urine by capillary electrophoresis with diode array and capacitively coupled contactless conductivity detection (CE-DAD-C^4^D)

**DOI:** 10.1007/s00216-026-06639-3

**Published:** 2026-07-06

**Authors:** L. A. A. Souza, S. M. Lunte, D. B. M. Weerasekara, M. A. Johnson, J. A. F. da Silva

**Affiliations:** 1 Instituto de Química, Universidade Estadual de Campinas, UNICAMP, Rua Monteiro Lobato 270, Campinas, SP 13083-862 Brazil; 2 Department of Chemistry, The University of Kansas, Irving Hill Road, Lawrence, KS 66044 USA; 3 Ralph N. Adams Institute of Bioanalytical Chemistry, The University of Kansas, Becker Drive, Lawrence, KS 66047 USA; 4 Department of Pharmaceutical Chemistry, The University of Kansas, Constant Avenue, Lawrence, KS 66047 USA; 5 Instituto Nacional de Ciência e Tecnologia em Bioanalítica Lauro Kubota, INCTBio-LK, Campinas, SP Brazil

**Keywords:** Dextroamphetamine, Capillary electrophoresis, Clinical analyses

## Abstract

Dextroamphetamine is a psychostimulant widely used in the treatment of attention-deficit/hyperactivity disorder and as a model compound in clinical toxicology and pharmacokinetic studies, and some studies report its use in cocaine dependence treatment. The present work describes the development and validation of a capillary electrophoresis (CE) method coupled with diode array (DAD) and capacitively coupled contactless conductivity (C^4^D) detectors for the simultaneous analysis of dextroamphetamine and its main urinary metabolites. Although phenylacetone is a key intermediate in the oxidative deamination pathway, this study focuses on the determination of 4-hydroxyamphetamine, norephedrine, hippuric acid, and benzoic acid in synthetic urine and spiked human samples. The method was optimized for background electrolyte composition, pH, and separation voltage, allowing efficient resolution and dual detection. Rapid separations were obtained, with migration times under 8 min. Linear responses were observed for all compounds within 1–200 μM (*R*^2^ ≥ 0.955), with LOD/LOQ ranging 0.32–32.2 μM and 1.07–97.6 μM, respectively. For dextroamphetamine, migration occurred at 3.64 min (DAD) and 3.41 min (C4D), with LOD/LOQ of 2.16/7.19 μM (DAD) and 8.58/28.60 μM (C4D). Intra- and inter-day repeatability tests demonstrated analytical precision with acceptable limits. The method was applied to spiked human urine samples using the standard addition technique for endogenous metabolites (hippuric acid and benzoic acid). This study demonstrates the feasibility of clinical and toxicological monitoring involving psychostimulant determination.

**Graphical abstract:**

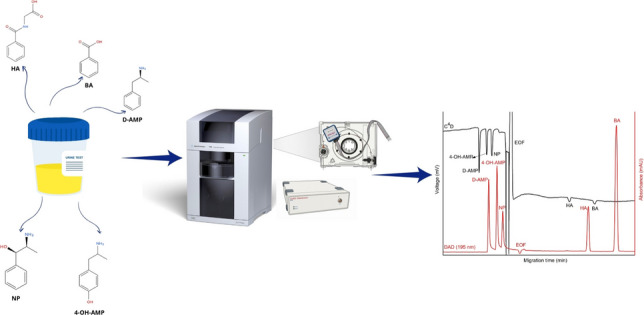

**Supplementary Information:**

The online version contains supplementary material available at 10.1007/s00216-026-06639-3.

## Introduction

 Dextroamphetamine (d-amphetamine) is a well-established central nervous system stimulant approved for the treatment of attention-deficit/hyperactivity disorder (ADHD) in several European countries [1, 2]. Furthermore, in light of the significant increase in global cocaine use—reaching record levels across regions including Europe, South America, North America, and Australia—the application of d-amphetamine as a potential agonist therapy for cocaine dependence has attracted growing biomedical interest, particularly regarding its use for reducing symptoms of stimulant use disorder [3–6]. Positive outcomes, including higher treatment adherence rates and reduced cocaine use, have been reported with doses between 15 and 60 mg, without serious adverse events. However, some studies have also demonstrated promising results with higher doses, with regimens of up to 120 mg per day being tested, highlighting the need to further evaluate appropriate dose adjustments for each case [3–7]. Given this therapeutic context, there is a critical need for robust, selective, and high-separation-efficiency bioanalytical methods capable of accurately monitoring d-amphetamine and its primary metabolites in human urine for therapeutic drug monitoring and compliance verification. In this regard, capillary electrophoresis (CE) offers several advantages, as it is a highly accessible alternative for implementation in resource-limited environments where access to liquid chromatography coupled with mass spectrometry (LC-MS) instrumentation may be restricted, while still providing reliable analytical sensitivity for monitoring therapeutic and toxicological stimulant levels.

The challenge in the bioanalysis of amphetamines is compounded by their complex metabolic profile and excretion kinetics. Amphetamines are primarily metabolized via two oxidative pathways: aromatic hydroxylation, mainly mediated by the CYP2D6 isoenzyme, yielding the pharmacologically active metabolite 4-hydroxyamphetamine [8, 9]. The other pathway is the oxidative deamination, catalyzed by CYP450 isoenzymes and flavin monooxygenase 3 (FMO3), initially producing inactive phenylacetone, which is subsequently oxidized to benzoic acid and then eliminated primarily as the hippuric acid conjugate [10–12]. Other key metabolites include norephedrine and phenyl-ketones. The stereoselectivity of this metabolism is critical, as d-amphetamine is metabolized faster than l-amphetamine, resulting in differing half-lives—approximately 8–10.5 h for d-amphetamine and 11–14 h for l-amphetamine—and leading to disproportionate amounts of the parent drug and its metabolites during elimination [13–15].

Urinary excretion is the primary route of elimination, but the excretion rate is highly dependent on urinary pH and flow: in acidic urine (pH 4.5–5.6), up to 57% of the unchanged compound may be excreted within 48 h, while in alkaline conditions (pH 7.1–8.0), excretion drops significantly to 7% [2, 16]. This pH dependency, coupled with the need for simultaneous separation and quantification of the parent drug and its active and inactive metabolites, presents a significant challenge for routine monitoring in both therapeutic and toxicological contexts. The quantitative analysis of d-amphetamine in urine is further complicated by its concentration variability, which is strongly influenced by metabolism, dose, and urinary pH. d-Amphetamine is frequently consumed directly or as part of an enantiomeric mixture, such as the prodrug lisdexamfetamine, which is extensively converted to d-amphetamine [17].

The excretion profile is complex: approximately 30% of a single d-amphetamine dose is excreted unchanged, with minor fraction excreted as active metabolites and the remainder as inactive metabolites [2, 9]. Given this extreme variability—where a 5 mg dose may result in concentrations below common screening cutoffs, and urinary pH can render interpretations of dose or timing unreliable—there is a need for analytical methods capable of reliably quantifying the parent drug and its major metabolites (e.g., 4-hydroxyamphetamine, norephedrine, and hippuric acid), as demonstrated in related amphetamine metabolism studies. In this context, portable CE-based approaches offer a practical alternative, particularly for resource-limited environments, even if they do not achieve the sensitivity typically provided by liquid chromatography coupled with mass spectrometry (LC-MS); providing a broad analytical range is still paramount for reliable clinical and toxicological monitoring [10, 18].

To overcome the limitations in throughput, high cost, and intricate sample preparation are often associated with gold-standard techniques such as gas chromatography coupled with mass spectrometry (GC-MS) and LC-MS/MS, CE emerges as a highly advantageous separation technique. CE offers several features suitable for bioanalysis, including high resolution and efficiency, fast analysis times, and low consumption of both sample and reagents, making it appropriate for analyzing drugs with stereochemical centers, such as d-amphetamine, and their metabolites. Furthermore, CE allows for the versatile coupling of various detection modes, including UV absorption with DAD, mass spectrometry (MS), laser-induced fluorescence (LIF), and electrochemical detection [18]. Among these options, capacitively coupled contactless conductivity detection (C^4^D) is particularly suitable for the analysis of charged analytes, as it operates non-invasively and does not require the optical transparency needed for UV detection [19–23]. The availability of commercial and open-source C^4^D systems further facilitates its integration into portable and resource-efficient analytical platforms, offering a powerful tool for monitoring charged analytes, like amphetamines and their ionic metabolites [24]. Additionally, the continuous advancements in C^4^D cell designs, microfluidic integration, and the implementation of dual detection systems have been comprehensively reviewed, highlighting the expanding capabilities of this technology in modern bioanalysis [25].

The present work reports, for the first time, the development of a dual detection using CE-DAD-C^4^D for the simultaneous separation and quantification of dextroamphetamine and its main metabolites (4-hydroxyamphetamine, norephedrine, hippuric acid, and benzoic acid) in human and synthetic urine samples spiked with the analytes of interest. This approach capitalizes on the advantages of DAD and C^4^D to provide a robust, orthogonal detection improving confidence in analyte identification for toxicological screening and monitoring of stimulant use.

## Experimental

### Reagents and solutions

Boric acid (Sigma-Aldrich) and sodium hydroxide (Fisher Scientific) were used to prepare the BGE. A 1.0 M sodium hydroxide stock solution was prepared by dissolving sodium hydroxide pellets (semiconductor grade, 99.99%, Sigma-Aldrich) in water. The following chemicals were obtained from Sigma-Aldrich (St. Louis, MO): d-amphetamine sulfate, hippuric acid, norephedrine hydrochloride, urea, uric acid, and ascorbic acid. Benzoic acid was purchased from Fisher Scientific (Fair Lawn, NJ). 4-Hydroxyamphetamine was obtained from Cayman Chemical (Ann Arbor, MI). Hydrochloric acid (Certified ACS Plus), methanol (LC/MS grade), and acetonitrile (HPLC grade) were obtained from Fisher Scientific. Deionized water (18.2 MΩ·cm) was produced using a Milli-Q Synthesis A10 purification system (Millipore, Billerica, MA). A 0.1 M hydrochloric acid solution was also prepared in DI water. Stock solutions of the analytes were prepared in DI water in 2000 μM, except for d-amphetamine, which was prepared at 1000 μM. All stock solutions were sonicated for 10 min in an ultrasonic bath and stored at 4 °C when not in use. BGE was prepared by dissolving boric acid to a final concentration of 30 mM, and the pH was adjusted to 9.20 using 1.0 M NaOH. Working standards were freshly prepared prior to CE analysis by diluting the stock solutions in BGE to the required concentration.

### CE-DAD-C^4^D

CE was performed using a G7100A CE instrument from Agilent Technologies equipped with a DAD detector. Fifty-micrometer inner diameter and 360-µm outer diameter Polymicro fused-silica capillaries (Fisher Scientific) were used. For CE-DAD-C^4^D experiments, the capillary length was 69.3 cm in total, with the C^4^D detector at 55 cm and the UV detection window at 60.8 cm. Windows for DAD detection were produced by burning off a section of polyimide using a CE window maker (Microsolv). Conditioning was accomplished using pressure at 70 mBar for 10 s and +20 kV, during which all wavelengths (195, 210, 220, 230, 250, 260, 265, and 270 nm) were monitored. However, calibration curves, limits of detection and quantification, and all reported results were determined exclusively at 195 nm. The BGE used for most experiments consisted of 30 mM boric acid, but several BGEs were evaluated to guarantee the best conditions for both detectors. A ER815 C^4^D detector system with a ET120 capillary headstage was used (eDAQ) for conductivity detection, inside of the cassette. The capillary was maintained at 25 °C and the optimized detection conditions were 750 kHz, 100% amplitude, range 0.05 V and 1 Hz low-pass filter. The limits of detection (LOD) and quantification (LOQ) were determined based on the signal-to-noise ratio (S/N) of 3 and 10, respectively. The baseline noise was measured in a region adjacent to the analyte peak in the electropherograms of a blank urine sample spiked at the lowest concentration level. The values were then calculated using the following equations: LOD = $3x ({h}_{\mathrm{n}\mathrm{o}\mathrm{i}\mathrm{s}\mathrm{e}}\backslash {C}_{\mathrm{s}\mathrm{l}\mathrm{o}\mathrm{p}\mathrm{e}})$ and LOQ = $10x ({h}_{\mathrm{n}\mathrm{o}\mathrm{i}\mathrm{s}\mathrm{e}}\backslash {C}_{\mathrm{s}\mathrm{l}\mathrm{o}\mathrm{p}\mathrm{e}})$; *h*_noise_ is the peak-to-peak noise and *C*_slope_ is the sensitivity from the calibration curve.

### Sample preparation

All individual stock solutions of the target analytes (dextroamphetamine sulfate (D-AMP), 4-hydroxyamphetamine (4-OH-AMP), norephedrine hydrochloride (NP), hippuric acid (HA), and benzoic acid (BA) were prepared in ultrapure water and subsequently diluted in the BGE. For initial method optimization, a commercial synthetic urine (Innovating Science) was diluted in BGE spiked with the target analytes to a final concentration of 100 μM for each compound, although various dilutions were initially evaluated. For validation and matrix effect assessment, a commercial pooled human urine matrix (Medix Biochemica) was employed (pH ≈ 6). Selectivity of the method was assessed by adding common endogenous urinary interferents (ascorbic acid, uric acid, urea, and creatinine) to synthetic urine solutions at physiological concentrations in the presence of the analyte standards. Commercial human urine was also spiked with the analytes and subjected to solid-phase extraction (SPE) using Pierce C18 Spin Columns (Thermo Scientific). The SPE consisted of cartridge conditioning with methanol and subsequent equilibration with a 5% acetonitrile aqueous solution containing 0.5% of trifluoroacetic acid (TFA). Then 100 µL of the spiked human urine sample was loaded, followed by a wash step with the wash solution (5% ACN/0.5%TFA) to remove interfering matrix components. Elution was performed using 200 µL of a methanol:acetonitrile (50:50 v/v) mixture. After that, the solutions were diluted in 30 mM borate BGE (pH 9.2) to final concentrations of 25, 50, and 100 μM for analysis by CE-DAD-C^4^D.

Repeatability (intra-day and inter-day precision) was assessed by analyzing a standard mixture at a concentration of 100 µM in BGE, with six injections performed on the same day over 3 days. This evaluation reflects the repeatability of the CE-DAD-C^4^D separation and detection, rather than the analysis of the urine matrix itself. Precision was expressed as the relative standard deviation (RSD). Method recovery was calculated by comparing the analyte response after the SPE protocol against the response from equivalent reference standards. These standards were prepared by spiking human urine with the analytes of interest (D-AMP, 4-OH-AMP, NP, HA, and BA) and diluting with BGE in appropriate proportions to simulate the expected concentrations of the final reconstituted eluate. Recovery assays were performed at 25, 50, and 100 µM for CE-DAD and 25 and 50 µM for CE-C^4^D. Additionally, to quantify the present hippuric acid and benzoic acid in the pooled urine, the standard addition method was employed. To quantify the endogenous hippuric acid and benzoic acid in pooled urine, aliquots of centrifuged and filtered urine (30 µL) were added to BGE. The samples were then spiked with HA and BA to achieve final concentrations of 25, 50, and 100 µM, and analyzed by CE-DAD.

## Results

**Fig. 1:**
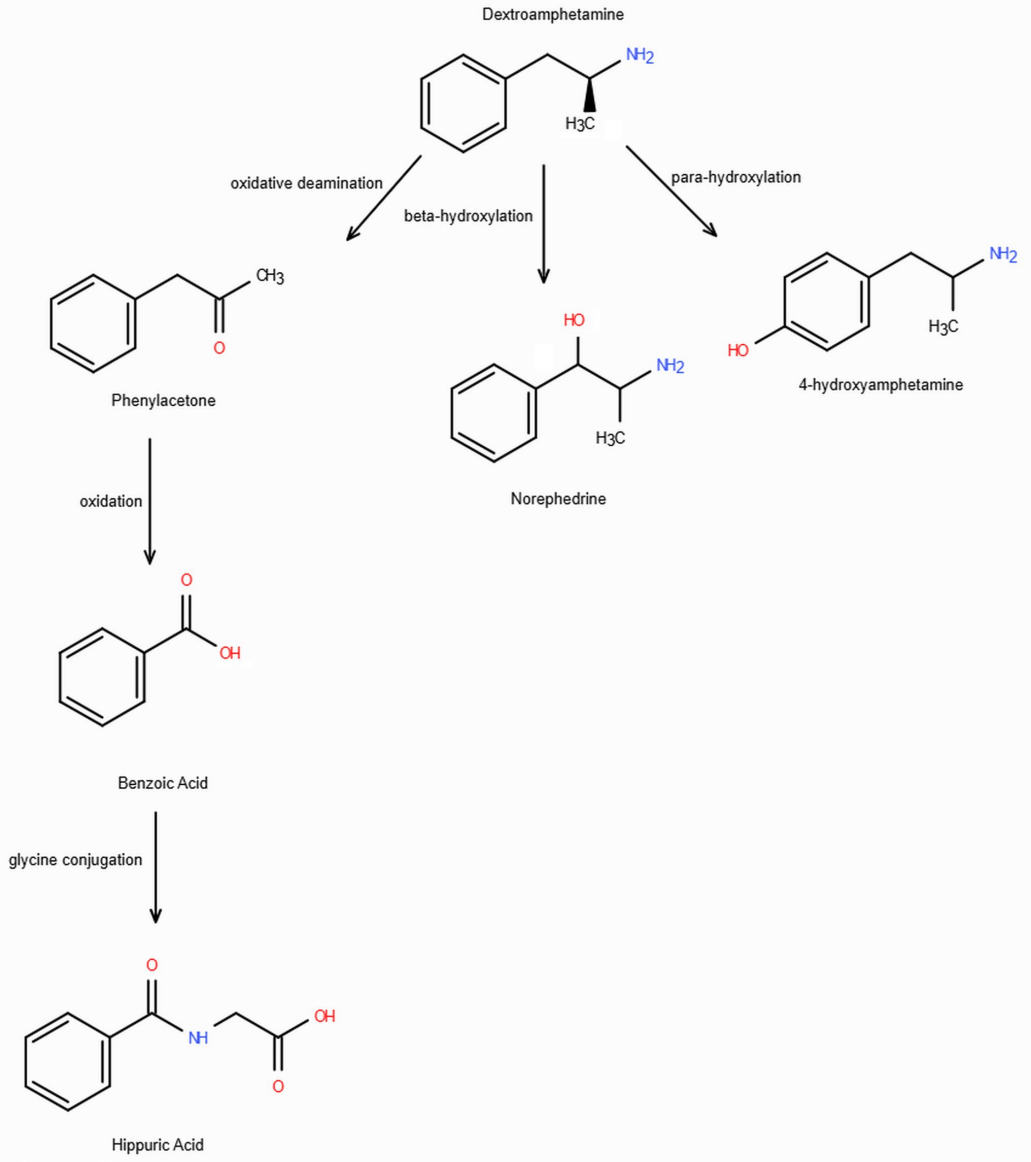
Main metabolic routes of dextroamphetamine. Sequential conversion into norephedrine (NP), 4-hydroxyamphetamine (4-OH-AMP), benzoic acid (BA), and hippuric acid (HA) via phase I and phase II (glycine conjugation) reactions (figure created with ChemDraw)

The method was developed for dextroamphetamine and its metabolites using CE-DAD-C^4^D. To evaluate the performance of the method for clinical applications, it was essential to target the primary metabolic products of dextroamphetamine. The biotransformation of dextroamphetamine in humans is complex, involving multiple enzymatic pathways that lead to both active and inactive metabolites. As illustrated in Fig. 1, the drug undergoes oxidative deamination to form phenylacetone, which is further oxidized to benzoic acid and subsequently conjugated with glycine to produce hippuric acid. Additionally, the molecule can undergo β-hydroxylation and para-conjugation, yielding norephedrine and 4-hydroxyamphetamine, respectively. In this study, except for phenylacetone, which is a transient intermediate, all major metabolites were selected as target analytes to ensure a comprehensive monitoring of the drug’s urinary profile.

As shown in the metabolic pathway (Fig. 1), the resulting analytes exhibit diverse physicochemical properties, including differences in *pKa* (D-AMP = 9.9, 4-OH-AMP = 9.5 (amine)/10.2 (phenol), NP = 9.4, HA = 3.6, and BA = 4.2) and UV absorption characteristics. For instance, while dextroamphetamine and norephedrine are basic compounds that remain cationic at the operating pH, benzoic and hippuric acids are acidic metabolites that migrate as anions. This chemical diversity justifies the implementation of a dual detection system: DAD was optimized for the aromatic rings of metabolites, while C^4^D provided a sensitive and universal response for the ionic species, particularly for those with low molar absorptivity.

The choice of a borate buffer at pH 9.2 as the BGE was strategic to ensure the simultaneous migration and resolution of all five compounds within a single run. Under these conditions, the electroosmotic flow (EOF) was sufficiently strong to elute the anionic species (BA and HA) towards the cathode, while maintaining adequate separation from the cationic parent drug and its hydroxylated metabolites.

### CE method optimization

A wavelength of 195 nm allowed the detection of the widest variety of target analytes. Boric acid was selected as the BGE because it demonstrated the best response for peak height in coupled analyses using DAD and C^4^D, although several other BGEs were tested for both detectors (Figures S1 and S2). Concentrations of boric acid (20, 25, 30, and 40 mM) were evaluated, and 30 mM was selected due to better resolution of certain species compared to other concentrations tested (Figure S3).

### Performance of the dual DAD-C^4^D detection

Additionally, C^4^D was evaluated. This method is based on differences in conductivity between the analyte and the BGE and does not require derivatization. As described in the Experimental section “CE-DAD-C^4^D,” the C^4^D parameters were optimized to provide sufficient detector response for all analytes, including benzoic acid and hippuric acid. In general, LOD and LOQ values obtained with C^4^D were higher than those obtained with UV detection (Table 2). Calibration curves showed good linearity, with *R*^2^ values greater than 0.95 across the concentration ranges evaluated.

**Fig. 2:**
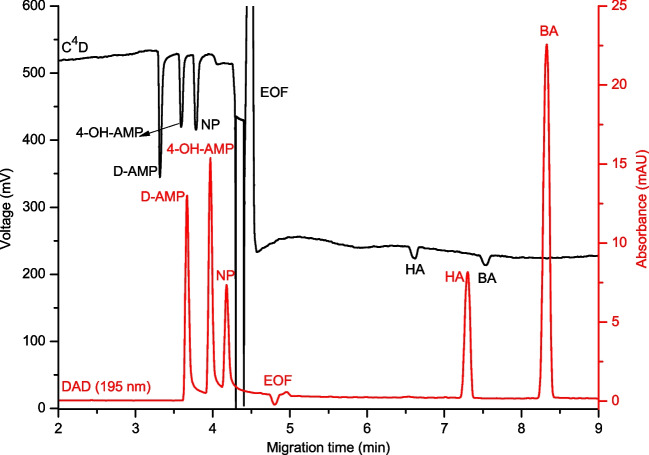
Separation of dextroamphetamine sulfate, 4-hydroxyamphetamine, norephedrine hydrochloride, hippuric acid, and benzoic acid with CE-DAD-C^4^D. Analyte concentrations, 100 µM. Conditions: capillary temperature, 25 °C; voltage, +20 kV; BGE, 30 mM borate buffer; buffer pH, 9.2. Injection, 70 mbar for 10 s. UV, 195 nm. C^4^D conditions, 750 kHz; full scale, 0.05 V; amplitude, 100%

The combination of C^4^D with DAD is shown in the electropherograms in Fig. 2. The developed method allowed the baseline separation of all analytes in less than 9 min. The selection of 30 mM borate buffer at pH 9.2 was primarily due to its ability to provide a low electrophoretic current (around 9 µA), which prevented capillary overheating and ensured a stable baseline. In contrast, other BGEs such as phosphate produced higher current, approximately five times increase, leading to baseline instability and poor C^4^D signal quality, despite yielding a satisfactory UV response. Additionally, the pH was optimized to ensure that all analytes remained predominantly ionized.

The electropherograms in Fig. 2 show that it was possible to perform the detection and separation of all analytes of interest using combined C^4^D and DAD detection. Table 1 gives the average migration times for the analytes at the two detectors.

**Table 1:** Migration times for dextroamphetamine and their main metabolites in CE-DAD-C^4^D

	**DAD**		**C**^**4**^**D**	
**Analyte**	**Average MT (min)**	**SD (min)**	**Average MT (min)**	**SD (min)**
D-AMP	3.64	0.01	3.41	0.01
4-OH-AMP	3.96	0.04	3.69	0.01
NP	4.09	0.01	3.87	0.01
HA	6.88	0.02	6.65	0.02
BA	7.75	0.02	7.54	0.04

*MT*, migration time; *n* = 6

As shown by the migration times in Table 1, the positively charged analytes—D-AMP, 4-OH-AMP, and NP—migrated first, followed by the negatively charged species, HA and BA, which migrated after the electroosmotic flow (EOF). The migration time for the EOF marker was 4.68 min for DAD and 4.60 min for C^4^D. The simultaneous detection and baseline separation of all analytes was achieved within 9 min. DAD provided selective detection based on the analytes’ chromophoric groups, while the universal C^4^D detector offered a conductivity-based response reflecting differences between ionic species and the background electrolyte. This dual detection approach demonstrates strong potential for toxicological analysis and the monitoring of stimulants in biological samples.

The use of two orthogonal detection systems (DAD and C^4^D) provides a significant advantage in complex matrices by combining spectral and conductivity-based information. This complementary approach enhances selectivity and reduces the risk of false positives from co-migrating interferents, which is particularly critical in biological samples such as urine. Furthermore, it enables reliable analyte confirmation as an accessible alternative to more complex and costly techniques such as mass spectrometry. The distinct response profiles of analytes in each detector further support the robustness of the method, contributing to improved confidence in both identification and quantification.

### Analytical validation

Calibration curves were obtained for both detectors for all analytes of interest, in the concentration ranges shown in Table 2.

**Table 2:** Calibration curve range and linear regression

	**DAD**			**C**^**4**^**D**		
**Analyte**	**Work range (µM)**	***R***^**2**^	**LOD (µM)**	**Work range (µM)**	***R***^**2**^	**LOD (µM)**
D-AMP	7.19–125	0.990	2.16	28.60–150	0.986	8.58
4-OH-AMP	8.43–125	0.991	2.53	15.51–150	0.998	5.12
NP	1.07–125	0.998	0.32	16.07–125	0.986	4.82
HA	1.73–100	0.998	0.57	97.6–175	0.955	32.2
BA	16.17–200	0.995	4.84	53.96–200	0.992	16.19

The lower limit of the work range corresponds to the limit of quantification (LOQ)

**Fig. 3:**
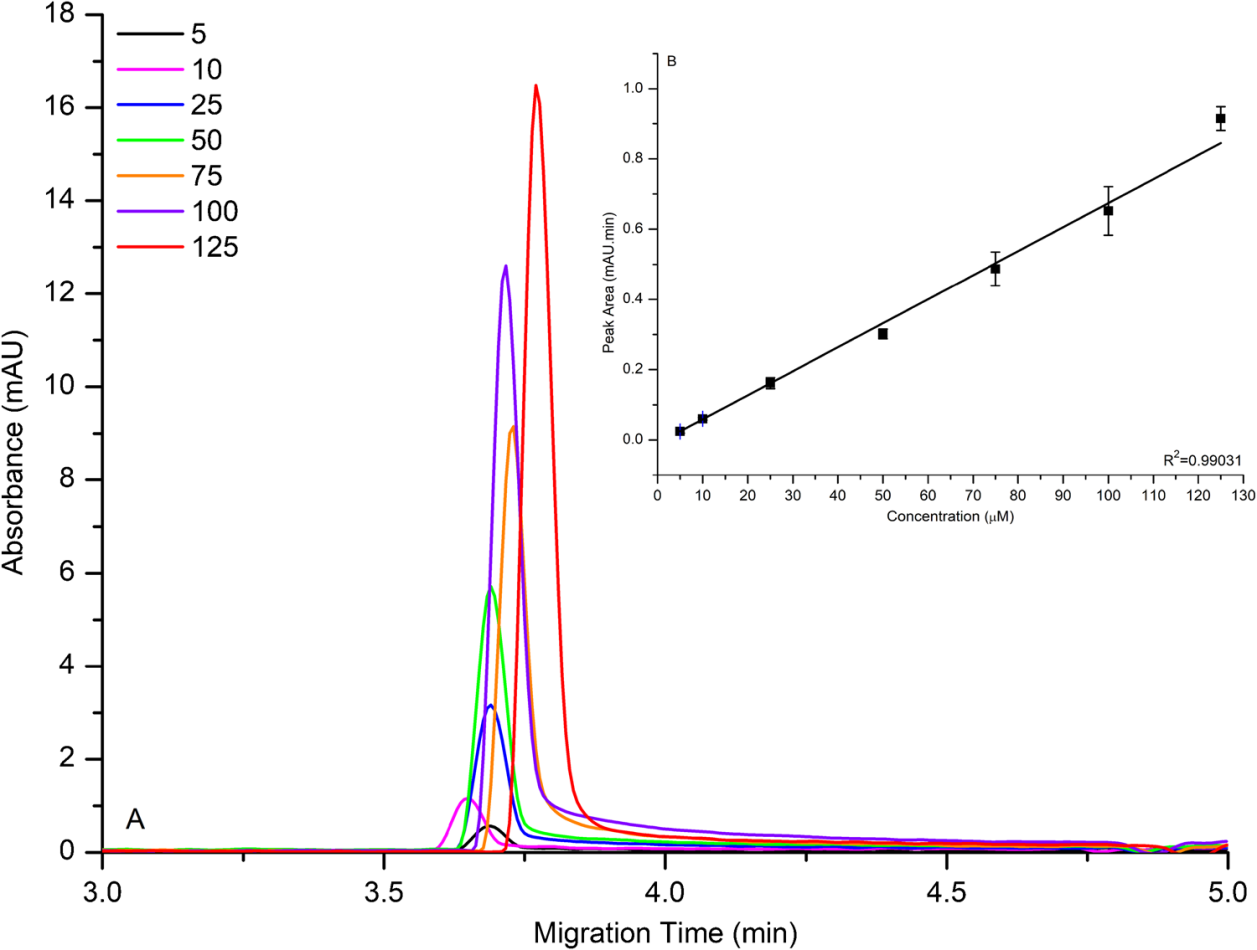
(**A)** Electropherogram for dextroamphetamine sulfate in CE-DAD with range 5–125 µM. Conditions: capillary temperature, 25 °C; voltage, +20 kV; BGE, 30 mM borate buffer; buffer pH, 9.2. Injection, 70 mbar for 10 s. UV detector, 195 nm. (**B**) Linearity of the UV detector responses for range 10–125 µM

**Fig. 4:**
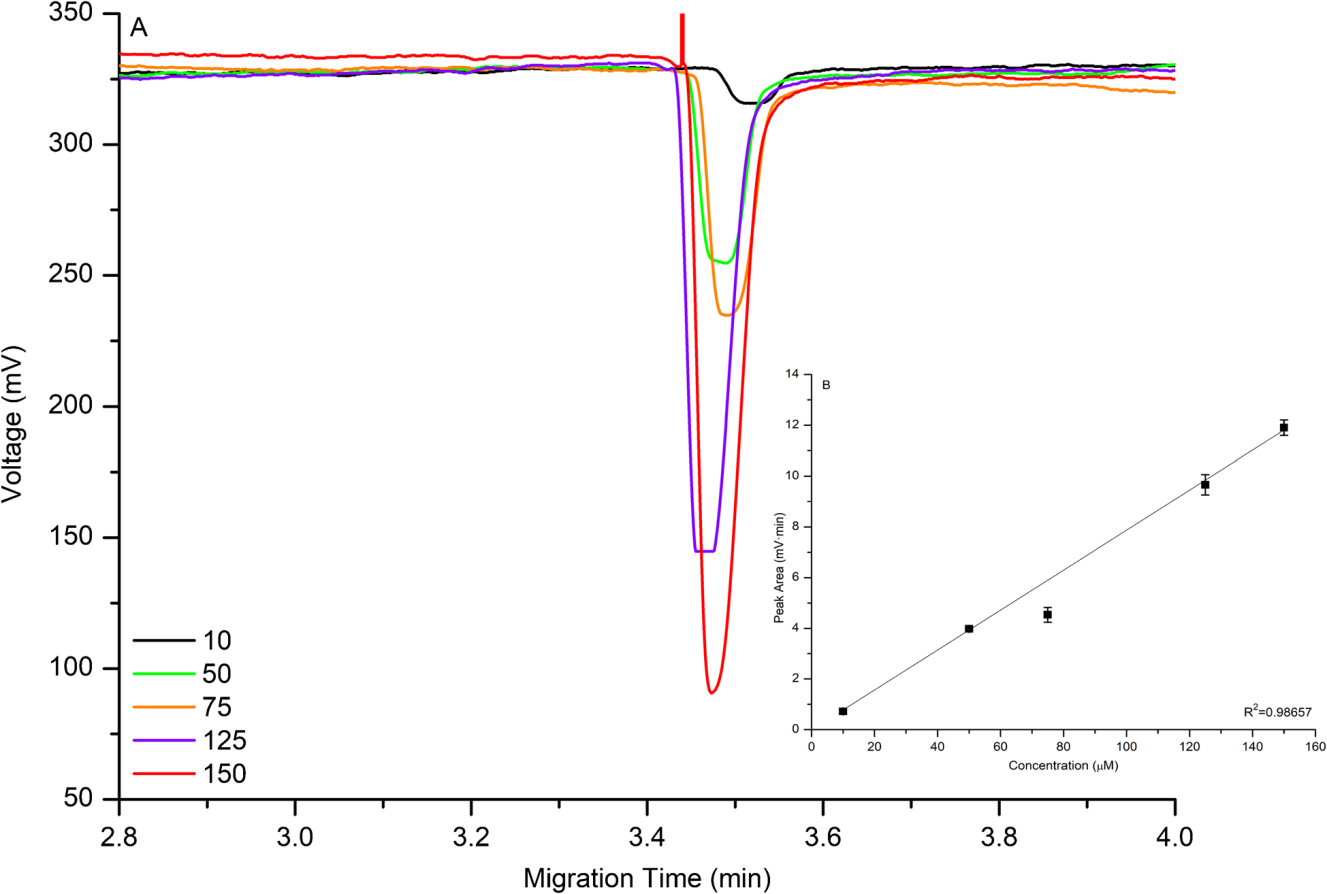
(**A**) Electropherogram for dextroamphetamine sulfate in CE-C^4^D with a range 10–150 µM. Conditions: capillary temperature, 25 °C; voltage, +20 kV; BGE, 30 mM borate buffer; buffer pH, 9.2. Injection, 70 mbar for 10 s. C^4^D conditions, 750 kHz; full scale, 0.05 V; amplitude, 100%; filter, 1 Hz. (**B**) Linearity of the C^4^D detector responses for a range 10–150 µM

The limits of detection and quantification were duly determined for all analytes of interest, using a minimum of 5 points, in triplicate, for the construction of the analytical curves. Figures 3 and 4 show the calibration curves and linear regression for dextroamphetamine using detection by CE-DAD-C^4^D.

The LOD and LOQ obtained for dextroamphetamine using DAD were 2.16 and 7.19 µM, respectively, and 8.58 and 28.60 μM using C^4^D. While our method is compatible with urinary levels reported by Torimitsu et al. (2023) who quantified amphetamine at 2970 ng/mL (approximately 22 μM) using LC-MS/MS, it provides sufficient sensitivity for clinical monitoring and therapeutic follow-up, where urinary concentrations are significantly higher [26]. Considering that therapeutic daily doses of d-amphetamine for attention-deficit disorder typically range from 10 to 30 mg, with approximately 30% excreted unchanged, the achieved limits are suitable for detecting these levels.

Regarding hippuric acid (HA), a key bioindicator of occupational exposure to solvents like toluene, ethylbenzene, and styrene, different analytical approaches have reported a wide range of LOD and LOQ values. Highly sensitive methods, such as those employing microextraction followed by HPLC-DAD (254 nm), achieved LOD and LOQ values as low as 0.15 µg/L (0.00084 μM) and 0.5 µg/mL (0.002789 μM), respectively, for HA in urine samples [27]. In another report using CZE-UV, the LOD was 0.21 mg/L (1.17 μM) and LOQ of 0.65 mg/L (3.63 μM), in this case hippuric acid as a biomarker of occupational exposure [28]. In comparison, our coupled detection system achieved an LOD of 0.57 μM and an LOQ of 1.73 μM for DAD, which demonstrates excellent sensitivity. Conversely, the C^4^D detector exhibited lower sensitivity, with LOD and LOQ values of 32.2 and 97.6 μM, respectively (Figure S6).

Benzoic acid was quantified in the study conducted by Lee et al. (2022) because it is one of the microbial-related metabolites associated with alterations in gut metabolic activity. Using a highly sensitive LC-MS/MS method, the authors reported a limit of quantification (LOQ) of 10–40 ng/mL (0.082-0.328 µM) for benzoic acid [29]. All calibration curves exhibited good linearity with a coefficient of determination (*R*^2^ > 0.98) for all analytes and detection modes, except for the benzoic acid calibration curve obtained via CE-C^4^D (Figures S4–S11). The UV spectra of each analyte in 30 mM borate buffer at pH 9.2 are presented in the Supplementary Material (Figures S16–S20).

In general, the DAD (195 nm) showed better overall sensitivity for all analytes evaluated, particularly hippuric and benzoic acids, compared to the C^4^D. Although calibration and primary results were calculated at 195 nm for optimal sensitivity, the CE-DAD method simultaneously monitored multiple wavelengths to confirm peak purity and identity. Electropherograms across all monitored wavelengths are provided in the Supplementary Material (Figure S21), clearly demonstrating the superior signal at 195 nm. Meanwhile, the C^4^D detector provides critical conductivity-based orthogonal information, ensuring unambiguous confirmation of analytes in complex biological matrices, where interfering compounds may comigrate or matrix effects may occur.

Intra-day repeatability (*n* = 6) and intermediate precision (*n* = 11), performed over two non-consecutive days, were assessed. Relative standard deviations (RSDs) were calculated for the corrected peak areas of all analytes. The results are summarized in Table 3, and the corresponding electropherogram overlays are provided in the Supplementary Material (Figures S12 and S13).

**Table 3:** Repeatability and intermediate precision (RSD%) of the analytical signals in background electrolyte (BGE)

	**DAD**		**C**^**4**^**D**	
**Analyte**	**%RSD** **Intra-day** **(** ${\boldsymbol{n}}$** =6)**	**%RSD** **Inter-day** **(** ${\boldsymbol{n}}$** =11)**	**%RSD** **Intra-day** **(** ${\boldsymbol{n}}$** =6)**	**%RSD** **Inter-day** **(** ${\boldsymbol{n}}$** =11)**
D-AMP	1.01	1.83	1.63	5.45
4-OH-AMP	1.23	1.80	1.56	5.21
NP	1.90	1.84	3.40	12.50
HA	2.30	2.22	4.59	10.20
BA	1.23	1.09	16.42	25.80

Separation of dextroamphetamine sulfate, 4-hydroxyamphetamine, norephedrine hydrochloride, hippuric acid, and benzoic acid with CE-DAD-C^4^D

Intra-day repeatability was assessed by analyzing six consecutive injections of the standard mixture (100 µM) in the background electrolyte (30 mM borate buffer, pH 9.2), calculating analytical peak areas for all analytes. Relative standard deviations (RSDs) obtained with the C^4^D detector were slightly higher than those from the DAD detector. This is likely due to system instabilities, as C^4^D is highly sensitive to temperature fluctuations and electronic noise in a modular setup. In general, intra-day variability was lower than intermediate precision (inter-day), as expected.

Intermediate precision was evaluated by repeating the procedure over two non-consecutive days (*n* = 11), using the same capillary and freshly prepared solutions. While inter-day variability was higher, migration times and peak areas remained consistent for the target analytes. Across all analytes, the highest RSD of peak areas was observed for benzoic acid with the contactless conductivity detector (25.8%). This specific result aligns with the lower linearity observed for this analyte in the conductivity mode, as previously discussed. Nevertheless, the target amphetamines (D-AMP and 4-OH-AMP) exhibited excellent intermediate precision, with RSDs below 5.5% in inter-day tests. These findings demonstrate that the method is robust and reliable for the separation and detection of the analytes of interest, with DAD providing superior quantitative performance while C^4^D offers valuable confirmatory data.

Repeatability was evaluated in BGE and is further illustrated in human urine for both DAD and C^4^D detectors (Supplementary Figures S14–S15).

### Application of the method to synthetic urine and spiked human urine

**Fig. 5:**
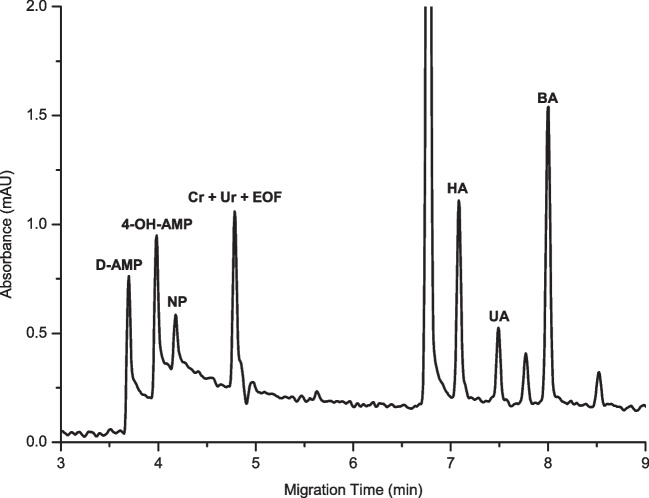
Test for majority interferents in urine sample (ascorbic acid, urea, uric acid, and creatinine) with dextroamphetamine sulfate, 4-hydroxyamphetamine, norephedrine, hippuric acid, and benzoic acid using CE-DAD. Peak IDs/analyte concentrations, 100 µM (final concentration in the injected sample), which corresponds to a 10-fold dilution of the original urine matrix (1:10 v/v) using BGE as the diluent. Conditions: capillary temperature, 25 °C; voltage, + 20 kV; BGE, 30 mM borate buffer (pH = 9.2). Injection, 70 mbar for 10 s. UV detector, 195 nm

To evaluate the applicability of the method for the detection of dextroamphetamine, 4-hydroxyamphetamine, norephedrine, hippuric acid, and benzoic acid in urine, the separation was first tested using synthetic urine spiked with the target analytes and compounds commonly present in urine, including ascorbic acid, uric acid, urea, and creatinine. The resulting electropherogram is shown in Fig. 5.

**Fig. 6:**
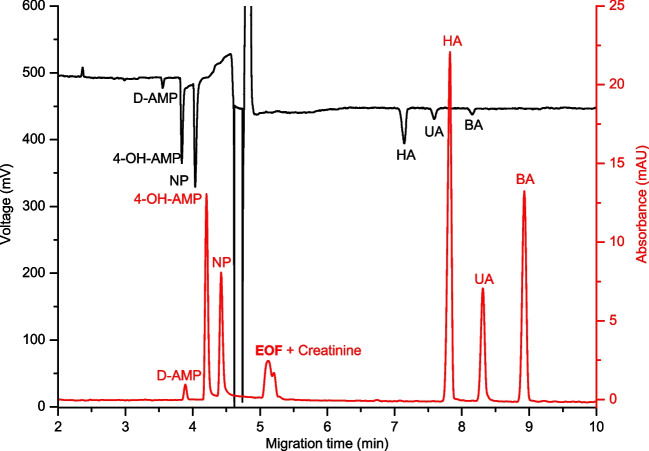
Test for majority interferents in urine sample (ascorbic acid, urea, uric acid, and creatinine) with dextroamphetamine sulfate, 4-hydroxyamphetamine, norephedrine, hippuric acid, and benzoic acid using CE-DAD-C^4^D. Peak IDs/analyte concentrations, 100 µM (final concentration in the injected sample), which corresponds to a 10-fold dilution of the original urine matrix (1:10 v/v) using BGE as the diluent. Conditions: capillary temperature, 25 °C; voltage, + 20 kV; BGE, 30 mM borate buffer (pH 9.2). Injection, 70 mbar for 10 s. C^4^D conditions, 750 kHz; full scale, 0.05 V; amplitude, 100%; filter, 1 Hz. UV detector, 195 nm

As illustrated in Fig. 5, the DAD exhibited selectivity towards potential interferents, including ascorbic acid, creatinine, uric acid, and urea. At the pH employed, creatinine and urea are neutral and migrated with the electroosmotic flow (EOF). The method was then applied to a commercially available certified human pooled urine. The matrix was spiked with the target analytes and potential interferents to yield a final concentration of 100 µM in the prepared sample after all processing steps. Solid-phase extraction (SPE) was used to isolate the analytes of interest and clean the sample, making it possible to evaluate both the CE method and the recovery efficiency of the extraction process. The corresponding results are presented in Fig. 6.

As illustrated in Fig. 6, the dual detection method allowed the detection and separation of all analytes of interest in this study, even in the presence of some endogenous compounds commonly present in human urine (ascorbic acid, uric acid, and urea). The total separation time was less than 10 min for both detectors. It is noteworthy that numerous studies focused on biomarker detection and urine analysis often monitor analytes such as uric acid, ascorbic acid, urea, and creatinine. Although creatinine, due to its low charge under the separation conditions, co-migrates with the electroosmotic flow (EOF) zone (as shown in the electropherogram with its pure standard), its detection by DAD is confirmed. Creatinine is particularly relevant, as its relatively constant excretion rate allows its use as a normalization factor for urinary analytes in pharmacokinetic studies [30–33]. In this study, the detection of dextroamphetamine and its related metabolites is reported for the first time using combined C^4^D and DAD, providing a new low-cost and selective analytical approach for urine analysis.

Regarding the concentrations of these analytes in urine, previous studies using high-sensitivity MS-based methods have reported levels of d-amphetamine and its related compounds ranging from 0.015 to 32.43 µM in various biological matrices [26, 34]. While the present CE-DAD-C^4^D method does not reach trace-level sensitivity, its detection limits (LOD of 2.16 µM) are fully compatible with the higher concentrations typically encountered in therapeutic monitoring and toxicological screenings, thus demonstrating its suitability for analyzing biologically relevant levels in urine.

Based on available literature data on the quantification of dextroamphetamine in urine and considering that the usual therapeutic doses range from 30 to 60 mg/day, with approximately 30% excreted unchanged, the compound is expected to be present in urine at concentrations between 30 and 120 µM. It should be noted that in studies addressing cocaine dependence treatment, higher doses of 120 mg or more may be administered, resulting in urinary concentrations above 150 µM. Therefore, the developed method is suitable for application to the analysis of urine samples, covering both the usual therapeutic range for attention-deficit disorder and higher concentrations associated with psychoactive-dependence treatments, as reported for cocaine [35, 36]. Samples exceeding the upper limit of the analytical range (125 µM) can be appropriately diluted to fit the calibration curve.

To evaluate the efficiency of the solid-phase extraction (SPE) process, a recovery test was performed using a spiked urine mix at concentrations of 25, 50, and 100 µM for both detectors, both before and after extraction. Table 4 presents the recovery values obtained for all analytes at the tested concentrations, using the CE-DAD-C^4^D detection method.

**Table 4:** Recovery test for CE-DAD-C^4^D

	**DAD**				C^4^D		
**Analyte**	**Recovery (%)** **25 µM**		**Recovery (%)** **50 µM**	**Recovery (%)** **100 µM**	**Recovery (%)** **25 µM**	**Recovery (%)** **50 µM**	**Recovery (%)** **100 µM**
D-AMP	61.9 ± 3.1		70.8 ± 5.1	94.6 ± 12.0	63.6 ± 17.4	98.4 ± 5.4	47.8 ± 18.8
4-OH-AMP	74.1 ± 5.1		65.35 ± 2.1	104.4 ± 4.2	88.5 ± 14.5	102.3 ± 2.9	19.5 ± 8
NP	68.2 ± 3.7		40.1 ± 5.8	99.9 ± 14.6	83.2 ± 7.5	91.1 ± 3.9	25.6 ± 6.5
HA	73.6 ± 7.6		48.4 ± 8.0	102.8 ± 4.2	94.7 ± 13.7	98.2 ± 8.2	116.1 ± 10.6
BA	49.8 ± 2.1		81.99 ± 7.6	89.99 ± 5.8	70.2 ± 21.7	102.8 ± 8.1	36.01 ± 1.8

The recovery validation of the CE-DAD-C^4^D method (Table 4) demonstrated variable efficiencies depending on the analyte’s physicochemical properties. d-Amphetamine (D-AMP) and 4-hydroxyamphetamine showed the most consistent results, with mean recoveries reaching up to 104.4% and precision with RSD < 15% for the majority of the DAD results, confirming the suitability of the SPE protocol for these target compounds. In contrast, the method showed limitations for the endogenous metabolites, hippuric acid (HA) and benzoic acid (BA). Values exceeding 100% for HA and BA in some levels do not necessarily reflect supra-recovery from the extraction, but rather the influence of their significant and variable endogenous concentrations in the human urine matrix. Additionally, the extraction efficiency monitored by C^4^D for HA and BA was compromised at lower concentrations, resulting in higher deviation values (RSDs of up to 35.4%) and reduced recoveries (e.g., 36.0% for BA at 100 µM in C^4^D). This variability reflects the high polarity of these metabolites, their low retention on C18 SPE cartridges, and the inherent heterogeneity of the biological matrix. Despite these restrictions for endogenous metabolites at specific levels, the combined SPE and CE-DAD- C^4^D method provides reliable recoveries for the primary psychoactive analytes. The target amphetamines exhibited RSD ≤ 15% in most tests, with recoveries within the acceptable range for complex bioanalytical matrices. Therefore, the protocol is suitable for clinical monitoring, maintaining robustness and precision for stimulant detection even in complex samples. By employing matrix-matched calibration, the method’s accuracy for the target drugs was maintained within acceptable limits for clinical screening.

### Determination of the concentration of HA and BA in the human urine sample

Hippuric acid and benzoic acid are frequently cited and investigated in urine analysis studies because they are common endogenous metabolites in humans. In this study, they were selected as model analytes to evaluate the method’s performance and selectivity in the presence of naturally occurring matrix components. Their concentrations can vary depending on diet, metabolism, and exposure to certain compounds, making them useful indicators for evaluating the performance and selectivity of analytical methods [37–39]. It is important to note that these compounds are not exclusive metabolites of dextroamphetamine and can be present endogenously in human urine due to various metabolic processes. This occurrence was confirmed in the commercially obtained human urine pool used in this study, and these compounds were subsequently quantified.

The separation and determination of hippuric acid (HA) and benzoic acid (BA) in urine are classically performed using high-efficiency chromatographic techniques, including gas chromatography (GC or GC-MS), high-performance liquid chromatography (HPLC or HPLC-MS), and CE, because these compounds are common endogenous metabolites and serve as important indicators for evaluating analytical method performance and selectivity [39]. In this study, BA and HA were detected in a human urine sample using the coupled detection system. Due to the endogenous nature of these analytes and the complexity of the urine matrix, quantification was performed using the standard addition technique to account for the non-zero baseline concentrations in the pool matrix. This approach allowed for the determination of the method’s accuracy in a “real-world” scenario where the blank matrix contains pre-existing analytes. Analyte standards (25, 50, and 100 µM) were added to the sample (Figure S23), while Figure S22 shows the blank human urine after filtration and centrifugation. The CE-DAD system determined endogenous concentrations of 63 ± 8 µM for HA and 57 ± 4 µM for BA. These results demonstrate that the method is selective enough to isolate and quantify these endogenous compounds without interfering with the analysis of the target amphetamines. The endogenous values quantified were evaluated against available literature data for non-exposed human urine (Table 5).

**Table 5:** Comparison of endogenous hippuric acid (HA) and benzoic acid (BA) concentrations in human urine, determined by different techniques

Study (reference)	Analyte	Reported concentration	Analytical technique
This study	Hippuric acid (HA)	63 ± 8 µM	CE-DAD
	Benzoic acid (BA)	57 ± 4 µM	CE-DAD
Damokhi et al. (2023) [41]	Hippuric acid (HA)	93.5–544.6 µM (NIOSH method 8301) 89.4–537.4 µM	DES-DLLME/HPLC-UV (230 nm)
Sioufi and Pommier (1980) [38]	Benzoic acid (BA)	818–6470 μM	GC-ECD
	Hippuric acid (HA)	894–6360 μM	GC-ECD

The endogenous concentrations of hippuric acid and benzoic acid determined in this work, as detailed in Table 5, are consistently at the lower end or significantly below the ranges reported by other studies (e.g., Damokhi et al. and Sioufi and Pommier) [38, 41]. This notable variation can primarily be attributed to a combination of sampling and methodological differences.

First, the nature of the sample is a critical factor. The concentrations obtained in the present study were determined using a certified, commercially acquired human urine mix. This type of pooled, quality-controlled material is typically employed as a standard reference and is likely to represent an average basal concentration from a non-exposed population, thus minimizing biological variance. In stark contrast, studies reporting wider and higher concentration ranges often analyze individual patient samples. This approach naturally captures significant biological variability, as the excretion of HA and BA is highly dependent on individual diet (ingestion of precursors) and metabolism. This incorporation of inherent human variability leads to the broader and higher maximum concentration observed in literature.

Second, methodological differences also contribute to the observed deviations. This study utilized CE-DAD, while comparative studies employed high-performance liquid chromatography with UV detection (HPLC-UV) or gas chromatography with electron capture detection (GC-ECD). The distinct specificity and sensitivity inherent to these analytical techniques can influence quantification, particularly in complex biological matrices or at low concentrations. Therefore, the combined use of a standardized, pooled reference sample and the CE-DAD technique is the primary reason why the determined HA and BA concentrations fall below those reported in studies that analyze variable individual samples using different analytical platforms.

## Conclusion

This work reports on the development and effective application of an advanced analytical technique for the monitoring of dextroamphetamine. For the first time, CE-DAD-C^4^D was utilized for the simultaneous analysis of dextroamphetamine and its metabolites (4-hydroxyamphetamine, norephedrine, hippuric acid, benzoic acid). The optimized CE-DAD-C^4^D method achieved complete and efficient separation of all five target analytes within an impressive total run time of just 10 min. The method offers rapid analysis times while maintaining high analytical reliability, making it suitable for routine toxicological applications. The integration of two orthogonal detection modes (DAD-C^4^D) proved highly advantageous for unequivocal compound confirmation in complex matrices, providing complementary information based on electrophoretic mobility and absorbance/conductivity.

This work reports on the development and effective application of an analytical technique for the monitoring of dextroamphetamine. For the first time, CE-DAD- C^4^D was utilized for the simultaneous analysis of dextroamphetamine and its metabolites (4-hydroxyamphetamine, norephedrine, hippuric acid, and benzoic acid). The optimized CE-DAD- C^4^D method achieved complete and efficient separation of all five target analytes within a total run time of 10 min. The method offers rapid analysis times while maintaining analytical reliability, making it suitable for routine toxicological applications. The integration of two orthogonal detection modes (DAD and C^4^D) proved advantageous for compound confirmation in complex matrices, providing complementary information based on electrophoretic mobility and absorbance/conductivity.

Furthermore, the obtained LOD and LOQ confirm the system’s sensitivity for tracking analytes within therapeutically and toxicologically relevant ranges, which is critical for monitoring patient compliance with stimulant therapy. The combination of efficient separation, rapid analysis, and complementary dual detection positions this method as a promising analytical tool for the clinical monitoring of dextroamphetamine. Moreover, this work establishes a foundation for future expansion into the broader detection of amphetamines in toxicological screening and controlled substance regulation applications. Given the simplicity of the CE platform and the inherent miniaturization capacity of the C^4^D detection system, this methodology holds significant promise for adaptation into low-cost, portable systems, enabling rapid and decentralized screening in resource-limited settings.

## Supplementary Information

Below is the link to the electronic supplementary material.

Supplementary file1 (DOCX 11.6 MB)

## Data Availability

The datasets generated and/or analyzed during the current study are available from the corresponding author on reasonable request.
