# Supplementary material for: Analyses of dextroamphetamine and its metabolites in human urine by capillary electrophoresis with diode array and capacitively coupled contactless conductivity detection (CE-DAD-C4D)
Source: Anal Bioanal Chem. 2026 Jul 6;418(15):4811–24. doi: 10.1007/s00216-026-06639-3 (PMC13388437; doi:10.1007/s00216-026-06639-3)
Supplement: Supplementary file 1 — Supplementary file1 (DOCX 11.6 MB) [file 216_2026_6639_MOESM1_ESM.docx]

**Analytical and Bioanalytical Chemistry**

**Electronic Supplementary Material**

Analyses of Dextroamphetamine and Its metabolites in Human Urine by Capillary Electrophoresis with Diode Array and Capacitively Coupled Contactless Conductivity Detection (CE-DAD-C^4^D)

Layla Amanda Acácio de Souza^1^, Susan M. Lunte^2,3,4^, Dhanushka Weerasekara^2,3^, Michael A. Johnson^2,3^, and José Alberto Fracassi da Silva^1,5^

^1^ Instituto de Química, Universidade Estadual de Campinas, UNICAMP, Rua Monteiro Lobato 270, Campinas, SP 13083-862, Brazil

^2^ Department of Chemistry, The University of Kansas, Irving Hill Road, Lawrence, KS 66044, USA

^3^ Ralph N. Adams Institute of Bioanalytical Chemistry, The University of Kansas, Becker Drive, Lawrence, KS 66047, USA

^4^ Department of Pharmaceutical Chemistry, The University of Kansas, Constant

Avenue, Lawrence, KS 66047, USA

^5^ Instituto Nacional de Ciência e Tecnologia em Bioanalítica Lauro Kubota, INCTBio-LK, Campinas, SP, Brazil
layla.souzacacio@gmail.com

##
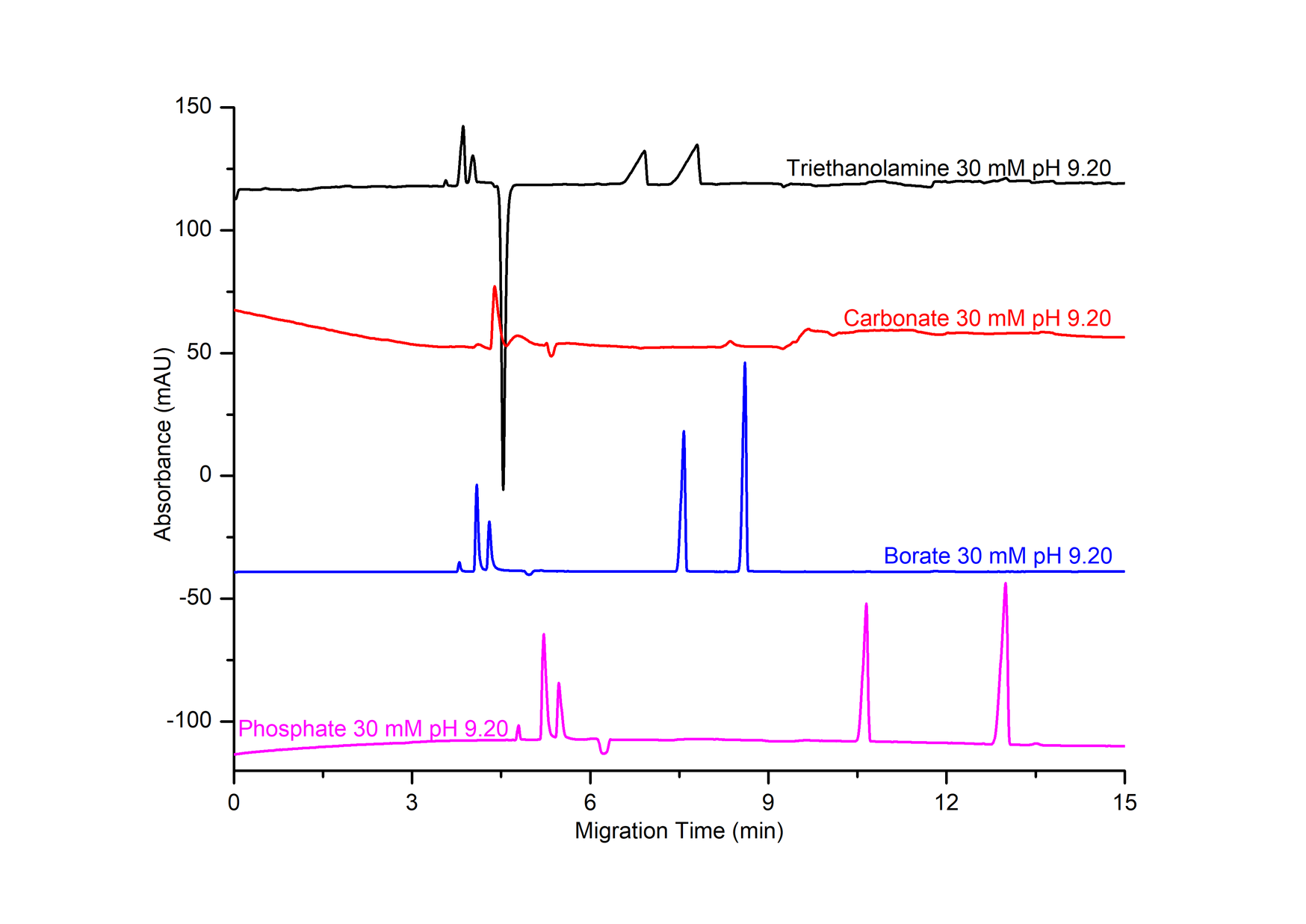


## Fig. S1 Other BGEs tested using 30 mM and pH=9.2 for dextroamphetamine sulfate, 4-hydroxyamphetamine, norephedrine hydrochloride, hippuric acid and benzoic acid with CE-DAD. Conditions: capillary temperature, 25 °C; voltage, + 20 kV; BGE, 30 mM borate buffer; buffer pH 9.2. Injection: 70 mbar for 10 seconds. UV: 195 nm.

The results presented in Figure S1 compare the impact of different Background Electrolytes (BGEs)—Triethanolamine, Carbonate, Borate, and Phosphate—all at 30 mM and pH 9.2, on the electrophoretic separation of a complex mixture containing three sympathomimetic amines (dextroamphetamine, 4-hydroxyamphetamine, and norephedrine) and two organic acids (hippuric acid and benzoic acid) for CE-DAD. In contrast to the Carbonate BGE, which exhibited the poorest selectivity and efficiency, resulting in co-migration of the analytes, the Borate and Phosphate BGEs provided the best separations. The Borate 30 mM (pH 9.2) was superior, offering the highest resolution and peak efficiency within a reasonable analysis time (8.5 min), suggesting that specific complex interactions of borate with 4-hydroxyamphetamine and norephedrine may be critical for optimizing selectivity. Although Phosphate also provided a four-peak separation, its analysis time was longer (after 12 min). It is concluded that Borate is the BGE of choice for this separation, providing the optimal foundation for the development of a robust analytical method, due to this this BGE was chosen to continue all the optimizations.

**
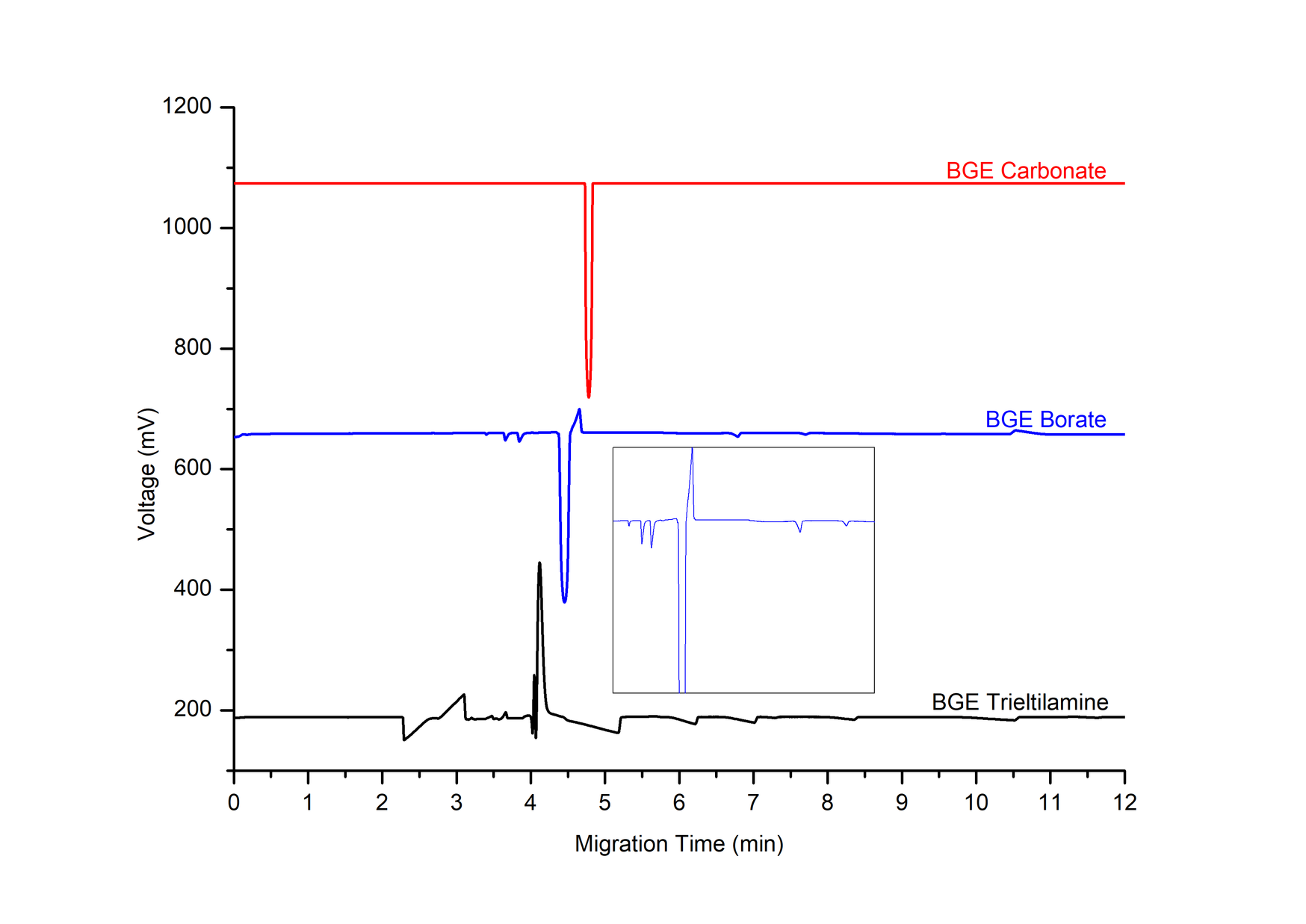
**

**Fig. S2** Other BGEs tested using 30 mM and pH=9.2 for dextroamphetamine sulfate, 4-hydroxyamphetamine, norephedrine hydrochloride, hippuric acid and benzoic acid with CE-C^4^D. Conditions: capillary temperature, 25 °C; voltage, + 20 kV; BGE, 30 mM borate buffer; buffer pH 9.2. Injection: 70 mbar for 10 seconds. UV: 195 nm. C^4^D conditions: 750 kHz, Amplitude 100%.

The selection of the Background Electrolyte (BGE) proved to be a critical factor due to the integration with Contactless Conductivity Detection (C^4^D). The Phosphate BGE (30 mM, pH 9.2) was excluded from subsequent optimization because its use resulted in an excessively high background current in the C^4^D (~ 50 µA), leading to an elevated risk of severe heating and potential detector damage. Consequently, optimization focused on the Borate, Carbonate, and Triethanolamine BGEs, which provided significantly lower and more stable background currents. Evaluating the electrophoretic performance, the Carbonate BGE yielded no signal response for any of the tested analytes, indicating a failure in separation and/or low ionic mobility. Triethanolamine, while showing initial signals, resulted in an unstable baseline and unsatisfactory detectability. In contrast, the Borate BGE (30 mM, pH 9.2) allowed for the observation of a response for all analytes, albeit initially with low signal intensity. Following instrumental adjustments (Full scale, amplitude, frequency and filter) to the C^4^D unit, which effectively increased the detector's sensitivity, a substantial enhancement in signal was achieved. This established Borate as the optimal BGE for the robust detection and quantification of all target analytes using contactless conductivity.

##
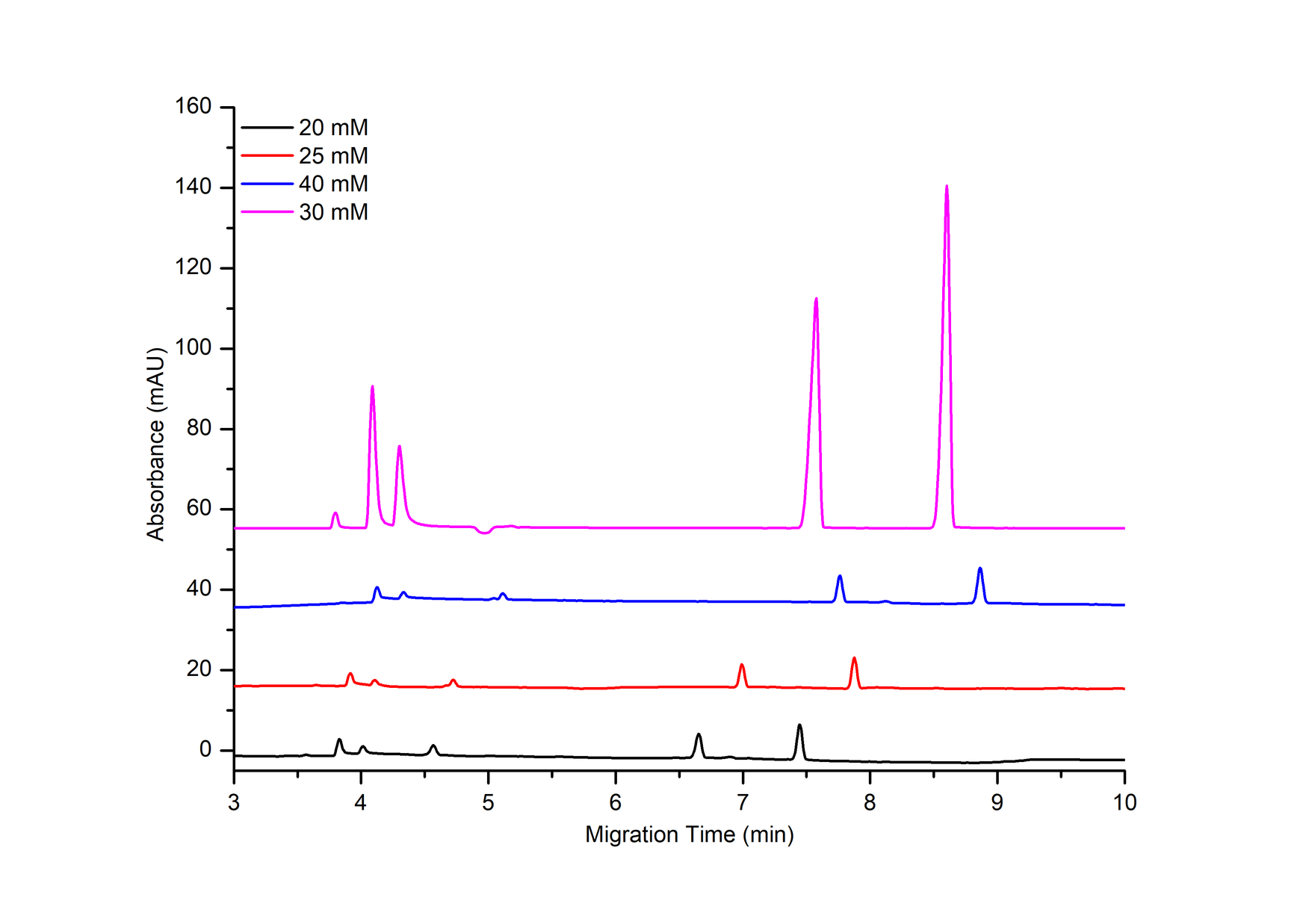


## Fig. S3 Optimization of BGE concentration for dextroamphetamine sulfate, 4-hydroxyamphetamine, norephedrine hydrochloride, hippuric acid and benzoic acid with CE-DAD. Conditions: capillary temperature, 25 °C; voltage, + 20 kV; BGE, 30 mM borate buffer; buffer pH 9.2. Injection: 70 mbar for 10 seconds. UV: 195 nm.

The influence of borate buffer concentration on the electrophoretic separation was investigated in the range of 20–40 mM. At lower concentrations (20–25 mM), the peaks were poorly resolved and showed lower signal intensity, indicating insufficient buffering capacity and reduced electroosmotic flow stability. Increasing the borate concentration to 30 mM improved the resolution between adjacent peaks and enhanced the signal intensity, providing sharper and more symmetric peaks. However, further increase to 40 mM resulted in broader peaks and longer migration times, probably due to higher current and Joule heating effects. Therefore, a concentration of 30 mM borate buffer at pH 9.2 was selected as the optimal BGE condition, providing the best compromise between resolution, migration time, and signal intensity.

**
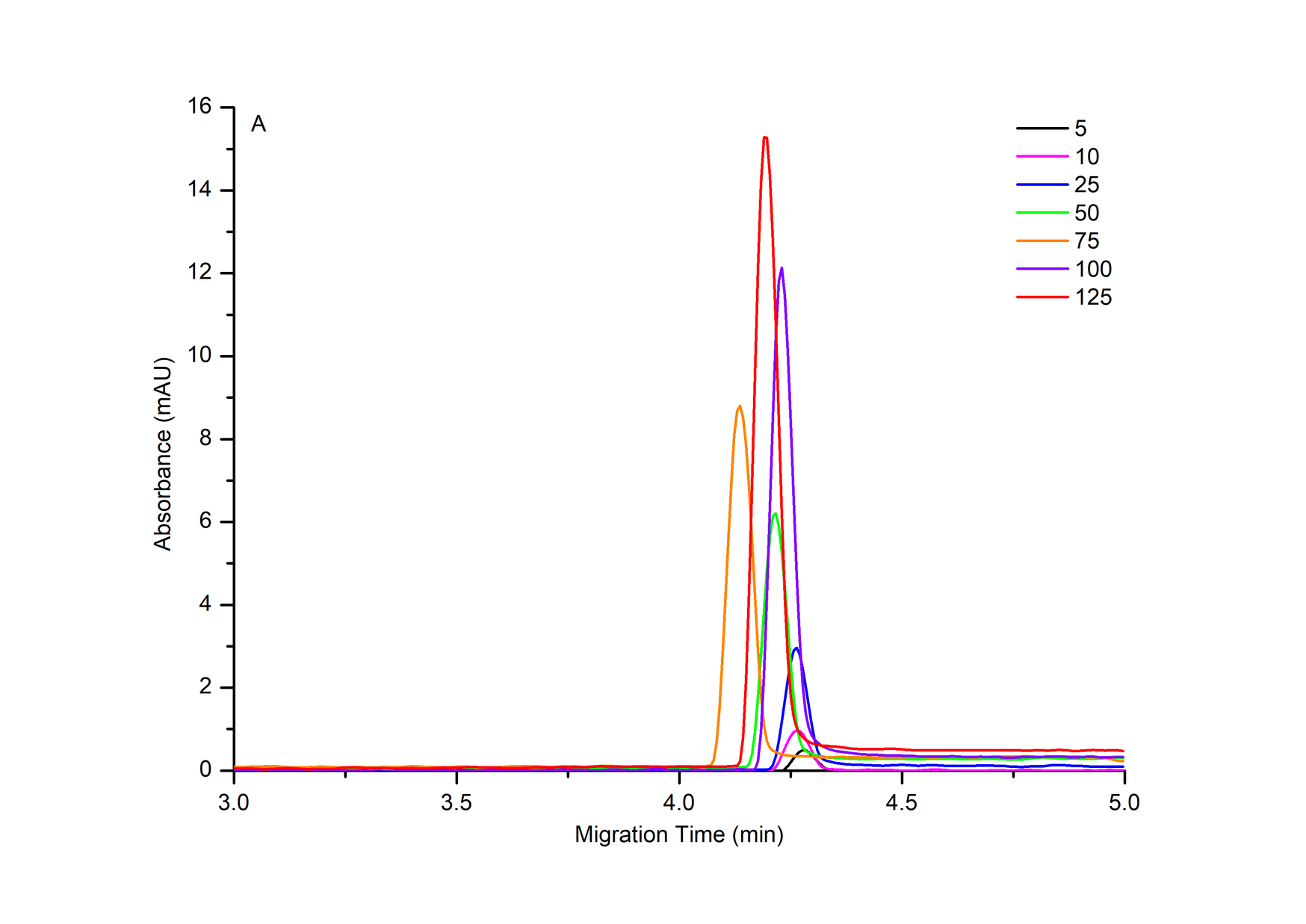

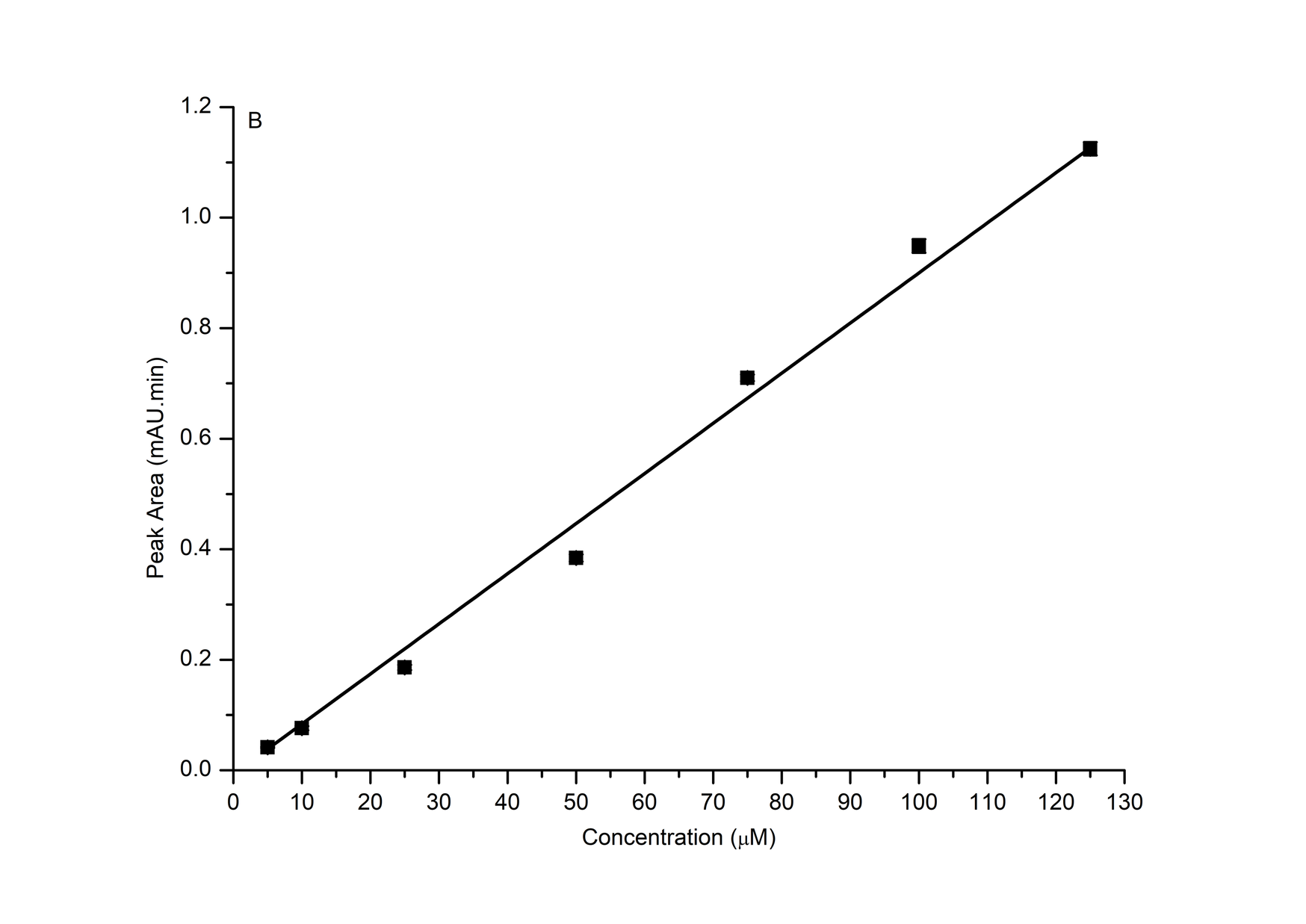
**

**Fig. S4** (A) Calibration curve for 4-hydroxyamphetamine in CE-DAD with range 5-125 µM. Conditions: capillary temperature, 25 °C; voltage, + 20 kV; BGE, 30 mM borate buffer; buffer pH 9.2. Injection: 70 mbar for 10 seconds. UV: 195 nm. (B) Linearity of the UV detector responses for range 5-125 µM, R^2^= 0.991.

The calibration curve for 4-hydroxyamphetamine was constructed using seven concentration levels in the range of 5–125 µM. The regression equation was obtained with a correlation coefficient (R²) of 0.991, demonstrating excellent linearity. Each concentration level was analyzed in triplicate, showing good repeatability of the signal.

**
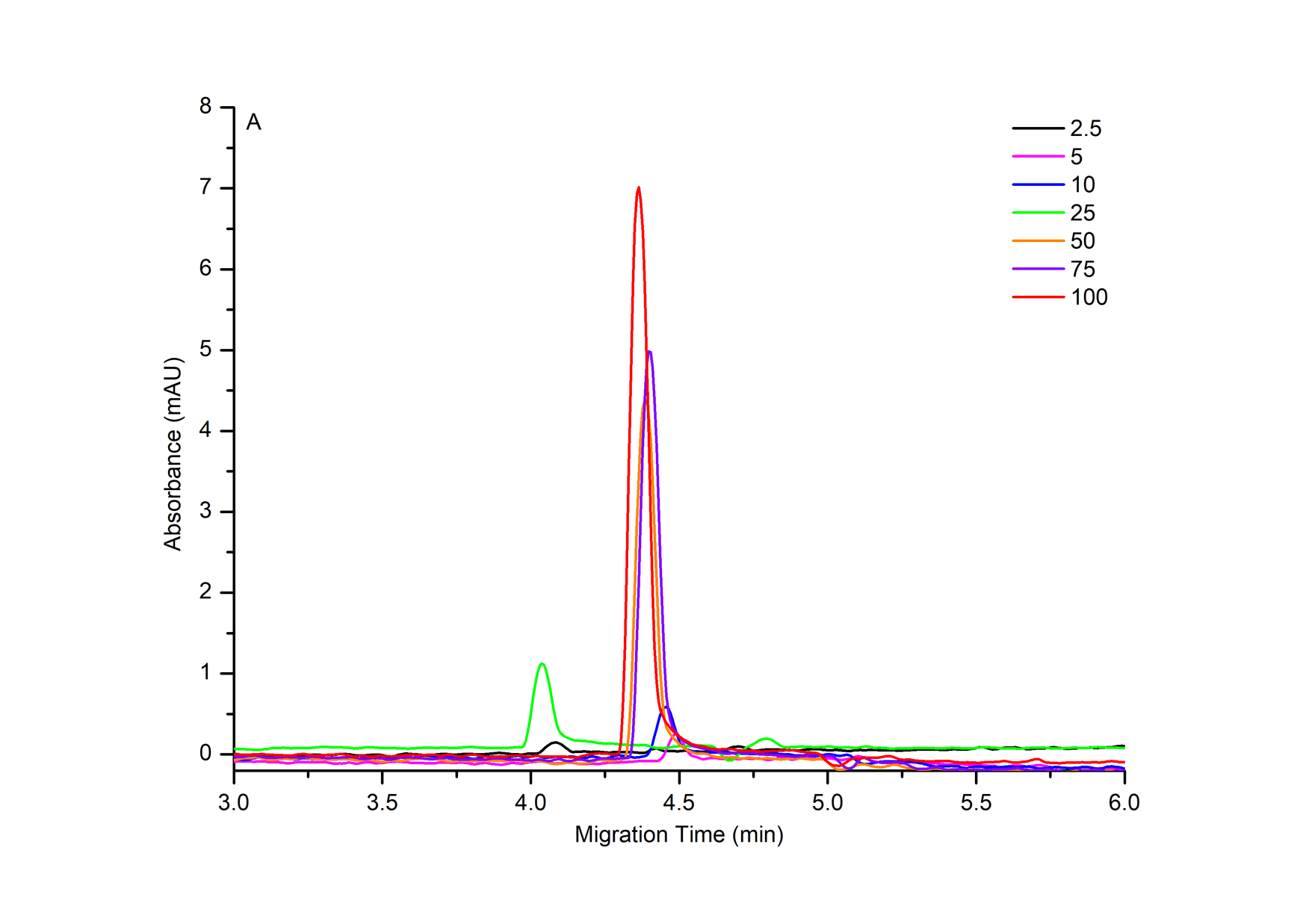
**

**
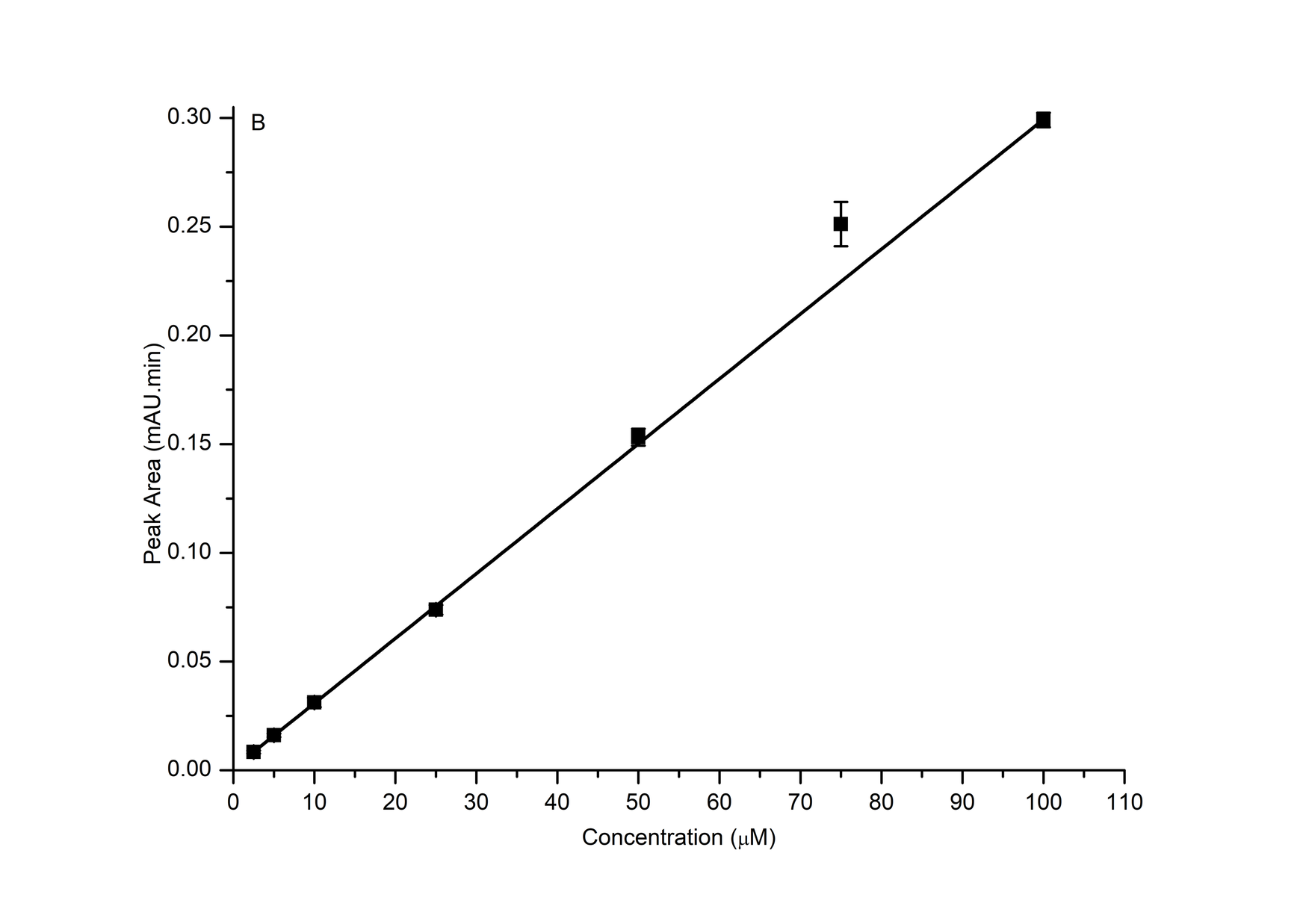
**

**Fig. S5** (A) Calibration curve for Norephedrine hydrochloride in CE-DAD with range 2.5-125 µM. Conditions: capillary temperature, 25 °C; voltage, + 20 kV; BGE, 30 mM borate buffer; buffer pH 9.2. Injection: 70 mbar for 10 seconds. UV: 195 nm. (B) Linearity of the UV detector responses for range 2.5-125 µM, R^2^= 0.998.

The calibration curve for norephedrine hydrochloride was constructed using seven concentration levels in the range of 2.5–100 µM. The regression equation was obtained with a correlation coefficient (R²) of 0.998, demonstrating excellent linearity. Each concentration level was analyzed in triplicate, showing good repeatability of the signal.

**
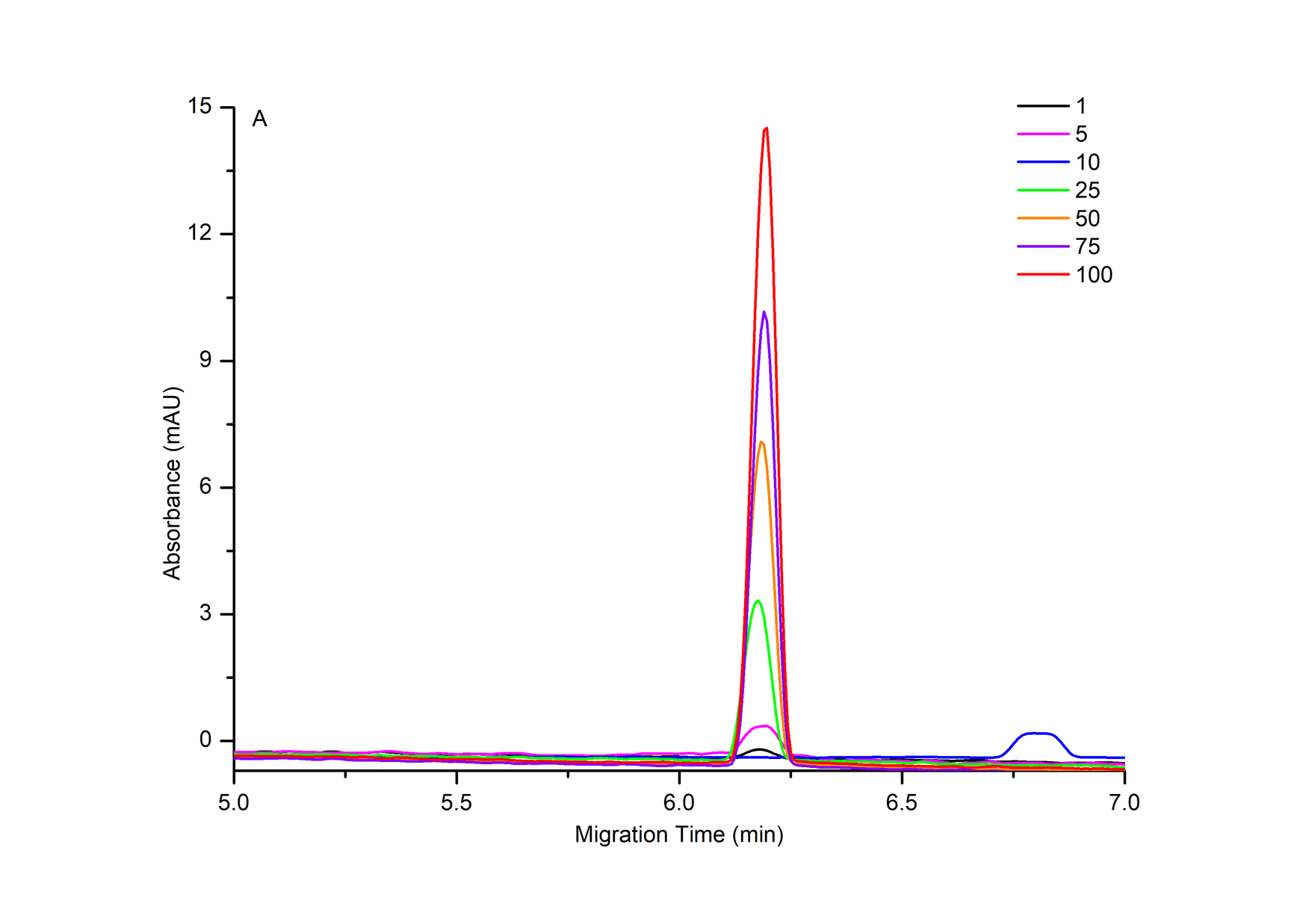

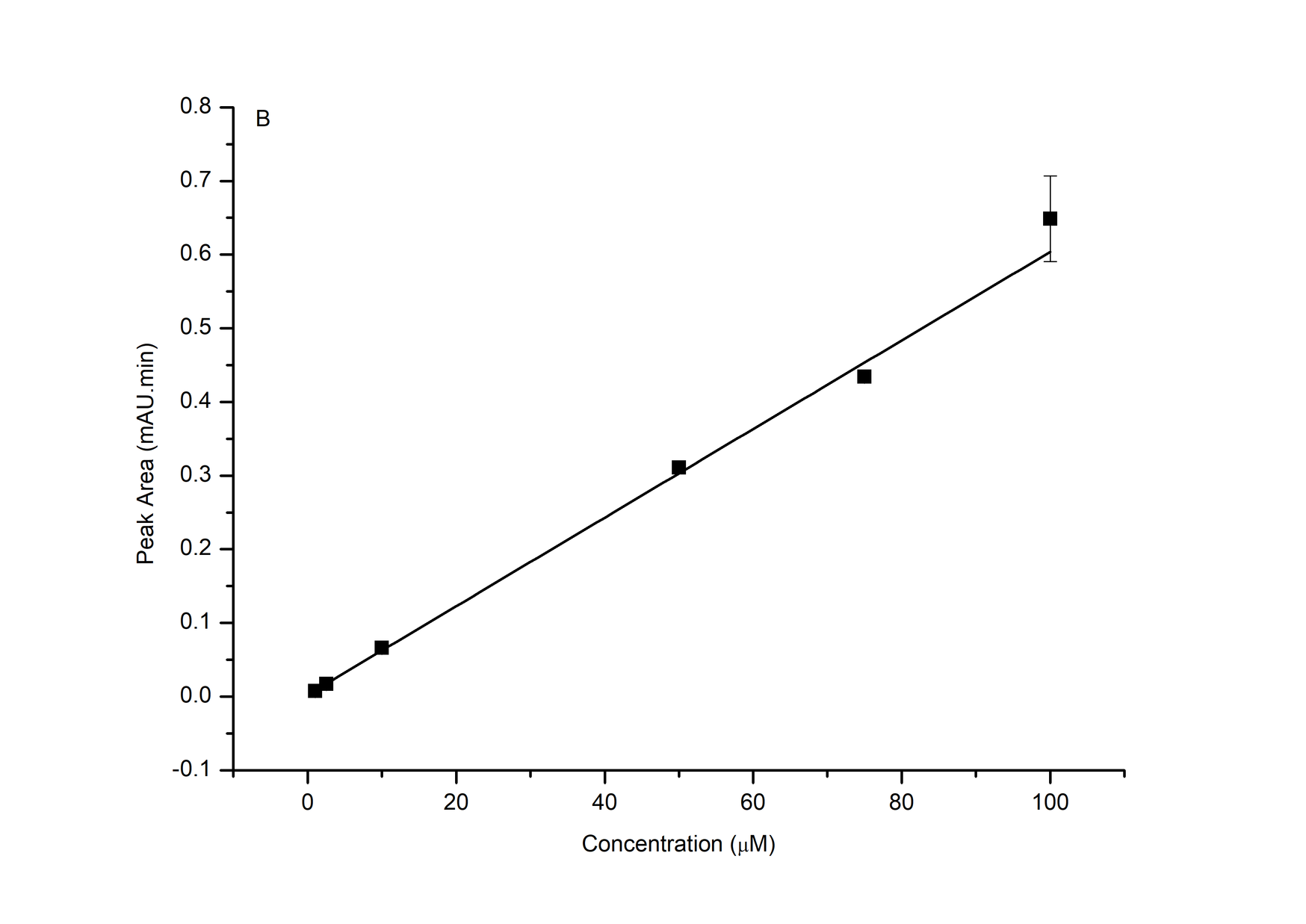
**

**Fig. S6** (A) Calibration curve for Hippuric Acid in CE-DAD within the range of 1.0–100 µM (*n* = 3). Conditions: capillary temperature, 25 °C; voltage, +20 kV; BGE, 30 mM borate buffer (pH 9.2). Injection: 70 mbar for 10 s. UV detection: 195 nm. (B) Linearity of the UV detector responses for the range of 1.0–100 µM (R^2^ = 0.998).

The calibration curve for hippuric acid was constructed using six concentration levels in the range of 1.0–100 µM. The regression equation was obtained with a correlation coefficient R^2^ of 0.998, demonstrating excellent linearity. Each concentration level was analyzed in triplicate (*n* = 3), showing good repeatability of the signal.

**
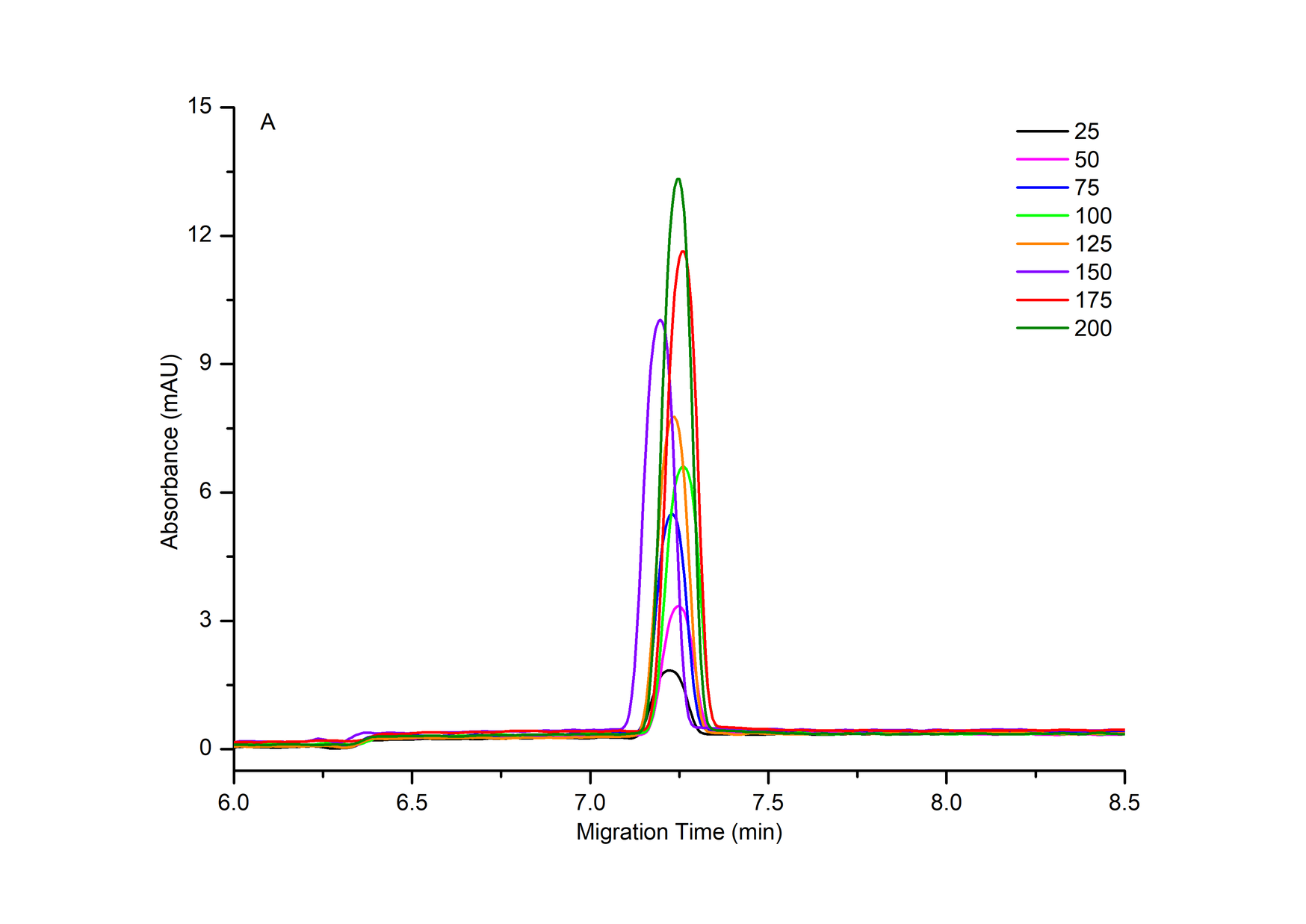

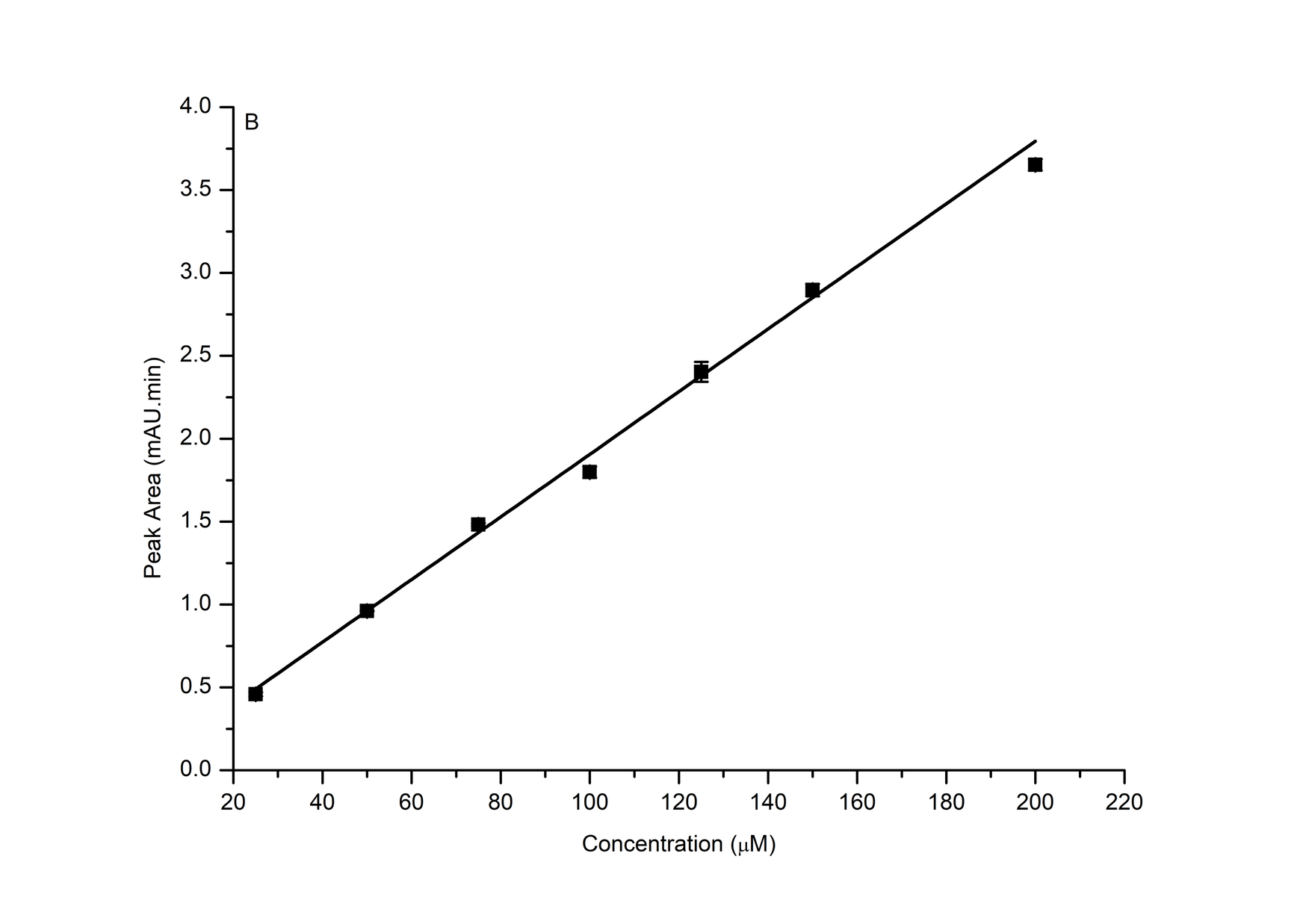
**

**Fig. S7** (A) Calibration curve for Benzoic Acid in CE-DAD within the range of 25–200 µM (*n* = 3). Conditions: capillary temperature, 25 °C; voltage, +20 kV; BGE, 30 mM borate buffer (pH 9.2). Injection: 70 mbar for 10 s. UV detection: 195 nm. (B) Linearity of the UV detector responses for the range of 25–200 µM (R^2^ = 0.995).

The calibration curve for benzoic acid was constructed using seven concentration levels in the range of 25–200 µM. The regression equation was obtained with a correlation coefficient (R^2^) of 0.995, demonstrating excellent linearity. Each concentration level was analyzed in triplicate (*n* = 3), showing good repeatability of the signal.

**
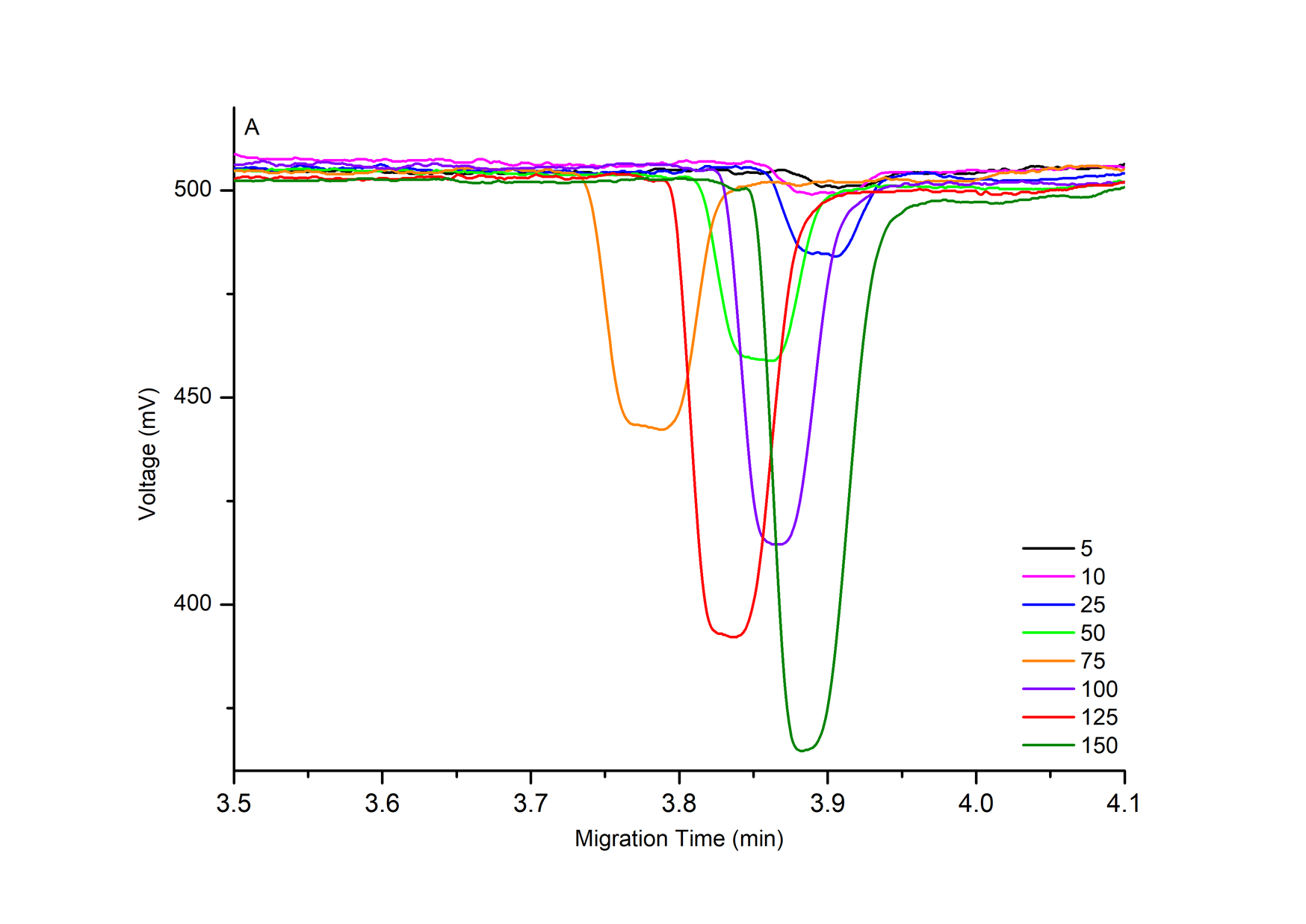

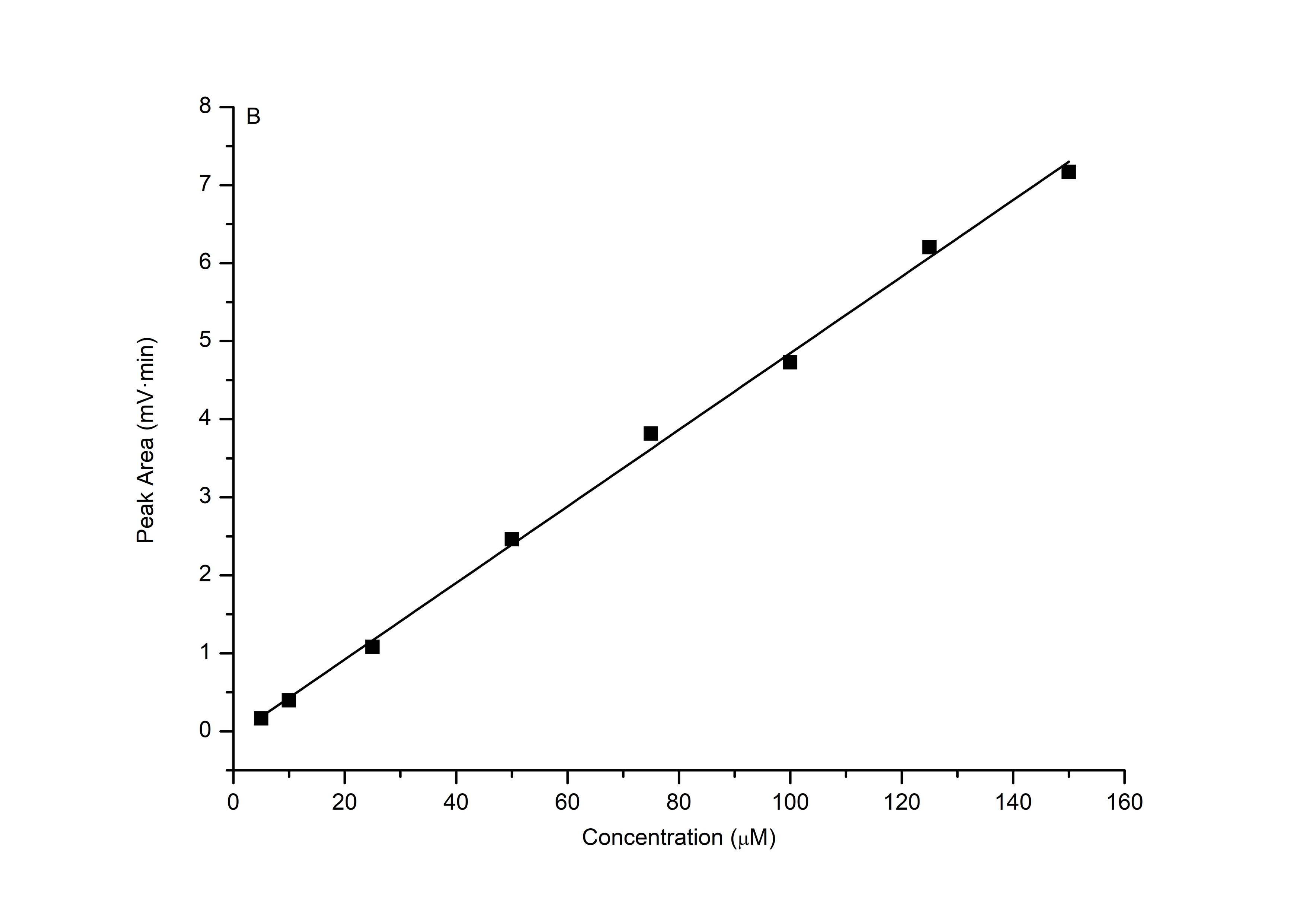
**

**Fig. S8** (A) Calibration curve for 4-hydroxyamphetamine in CE-C^4^D within the range of 5–150 µM (*n* = 3). Conditions: capillary temperature, 25 °C; voltage, +20 kV; BGE, 30 mM borate buffer (pH 9.2). Injection: 70 mbar for 10 s. C^4^D conditions: frequency 750 kHz, full scale 0.05 V, amplitude 100%, filter 1 Hz. (B) Linearity of the C^4^D detector responses for the range of 5–150 µM (R^2^ = 0.998).

The calibration curve for 4-hydroxyamphetamine was constructed using the concentration levels in the range of 5–150 µM. The regression equation was obtained with a correlation coefficient (R^2^) of 0.998, demonstrating excellent linearity. Each concentration level was analyzed in triplicate (*n* = 3), showing good repeatability of the signal.

**
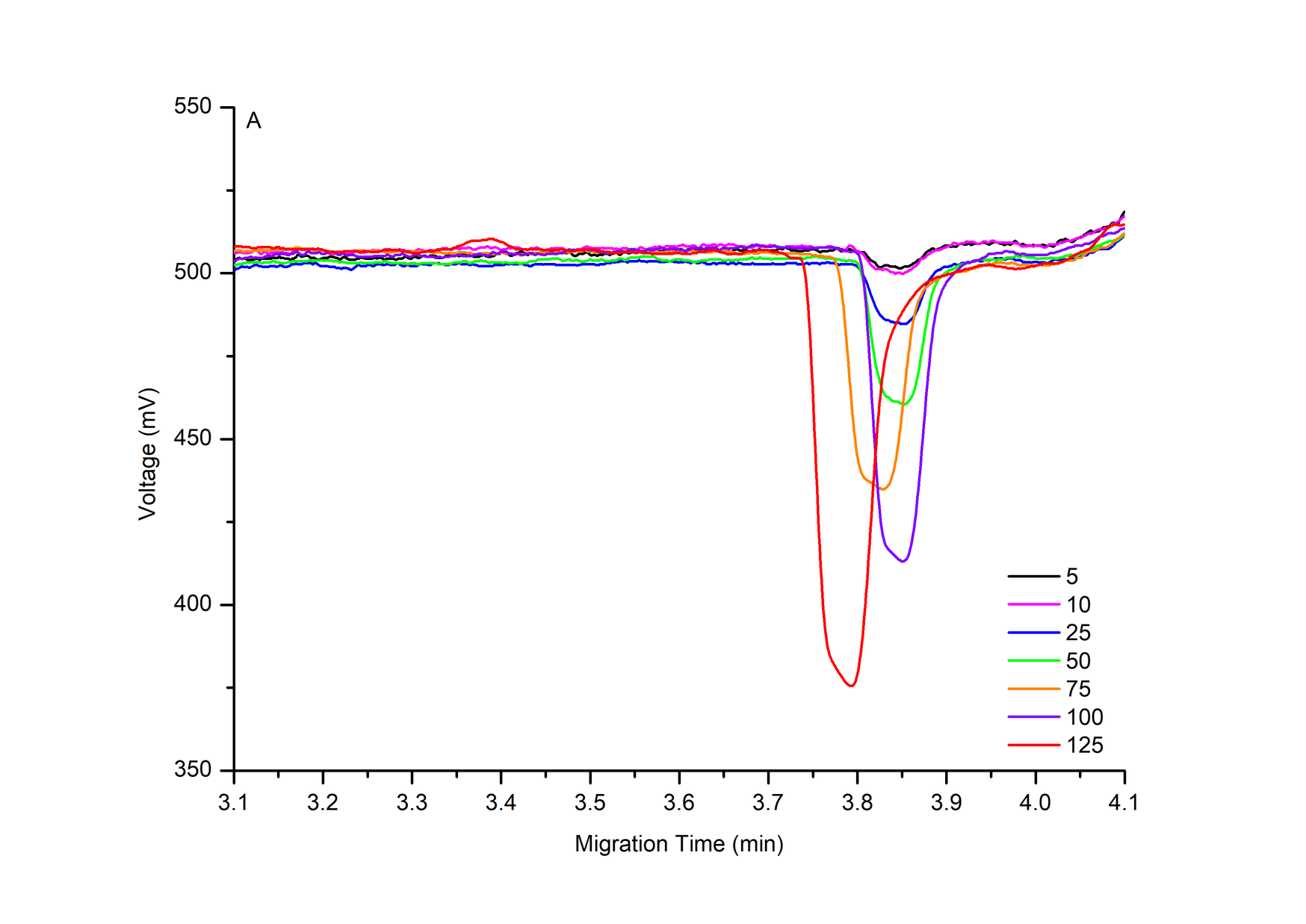

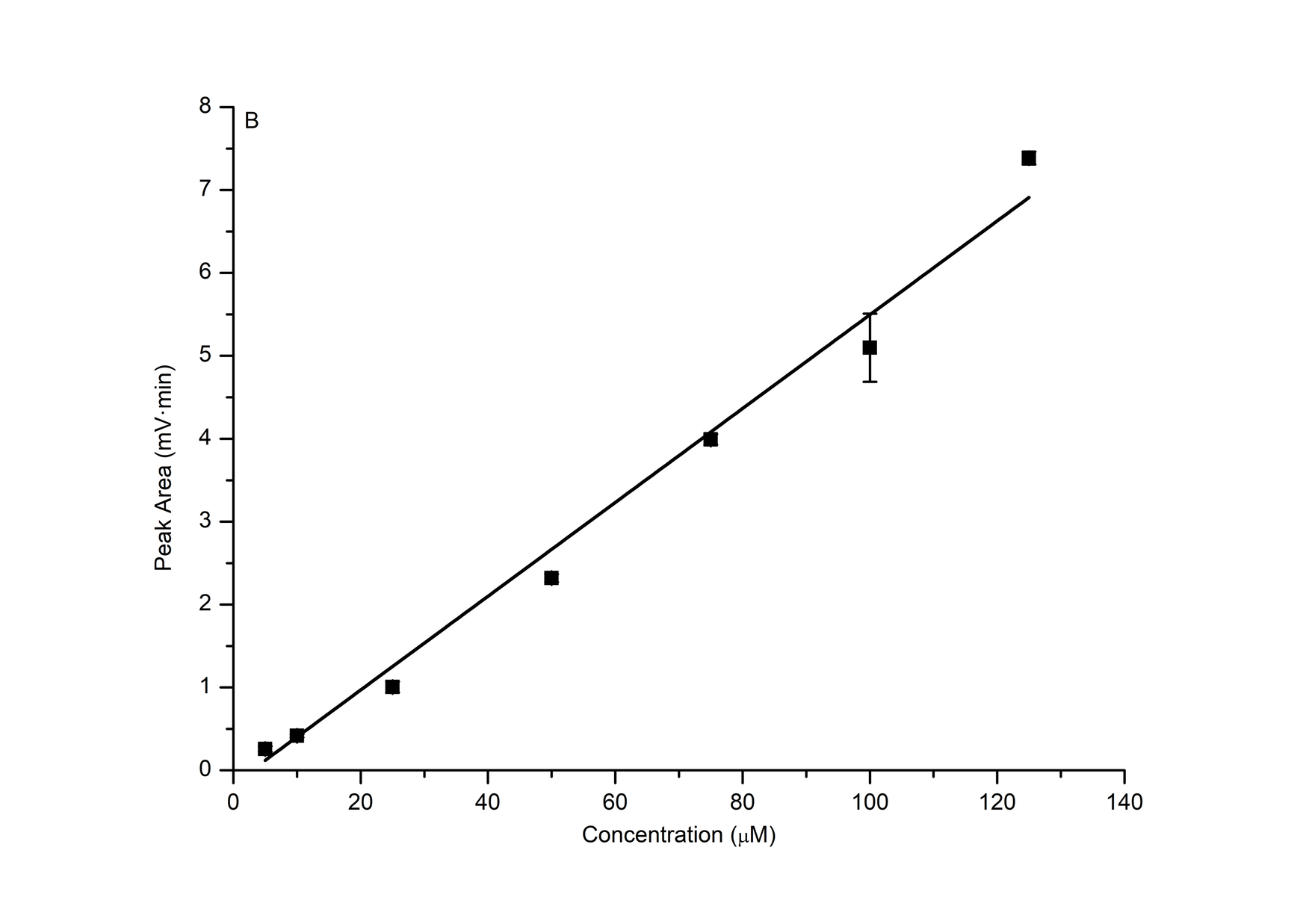
**

**Fig. S9** (A) Calibration curve for Norephedrine hydrochloride in CE-C^4^D with range 5-125 µM. Conditions: capillary temperature, 25 °C; voltage, + 20 kV; BGE, 30 mM borate buffer; buffer pH 9.2. Injection: 70 mbar for 10 seconds. C^4^D conditions: 750 kHz, Full scale 0.05 V, Amplitude 100%, Filter 1 Hz. (B) Linearity of the C^4^D detector responses for range 5-125 µM, R^2^=0,986.

The calibration curve for norephedrine hydrochloride was constructed using seven concentration levels in the range of 5–125 µM. The regression equation was obtained with a correlation coefficient (R²) of 0.986, demonstrating excellent linearity. Each concentration level was analyzed in triplicate, showing good repeatability of the signal.


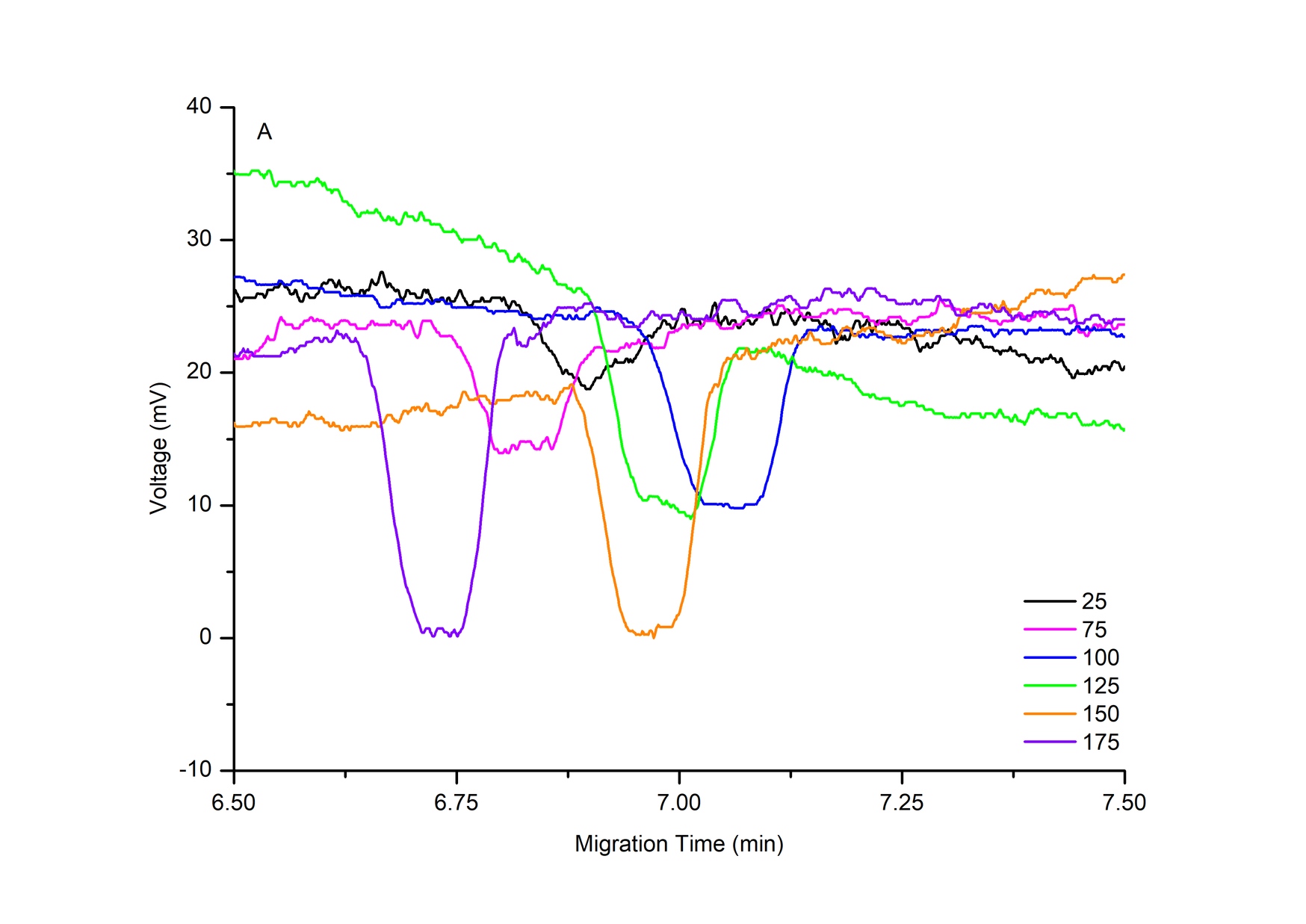

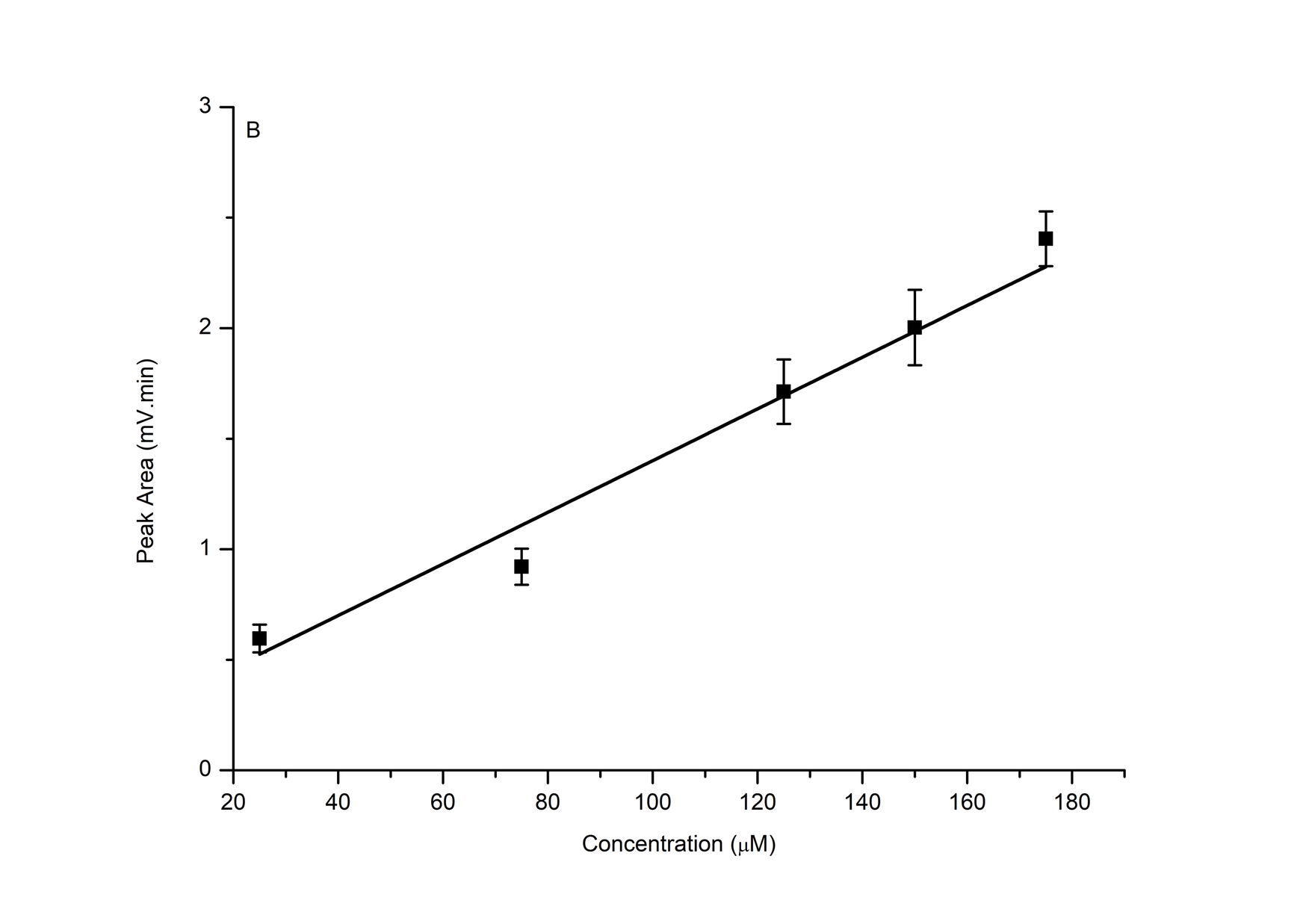


**Fig. S10** (A) Calibration curve for HA in CE-C^4^D with range 25-175 µM. Conditions: capillary temperature, 25 °C; voltage, + 20 kV; BGE, 30 mM borate buffer; buffer pH 9.2. Injection: 70 mbar for 10 seconds. C^4^D conditions: 750 kHz, Full scale 0.05 V, Amplitude 100%, Filter 1 Hz. (B) Linearity of the C^4^D detector responses for range 25-175 µM, R^2^=0,955.

The calibration curve for hippuric acid was constructed using five concentration levels in the range of 25-175 µM. The regression equation was obtained with a correlation coefficient (R²) of 0.955, demonstrating excellent linearity. Each concentration level was analyzed in triplicate, showing good repeatability of the signal.


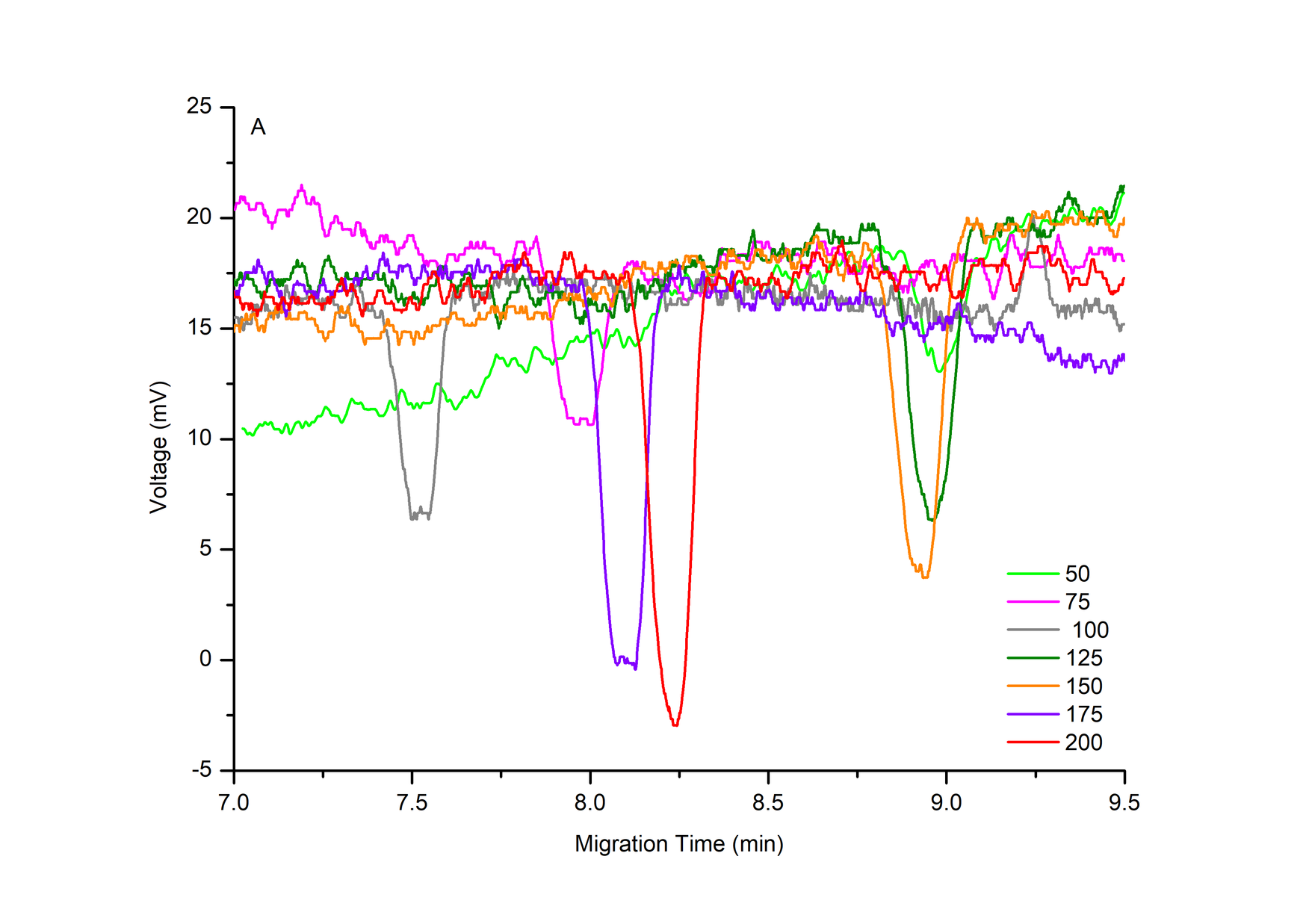

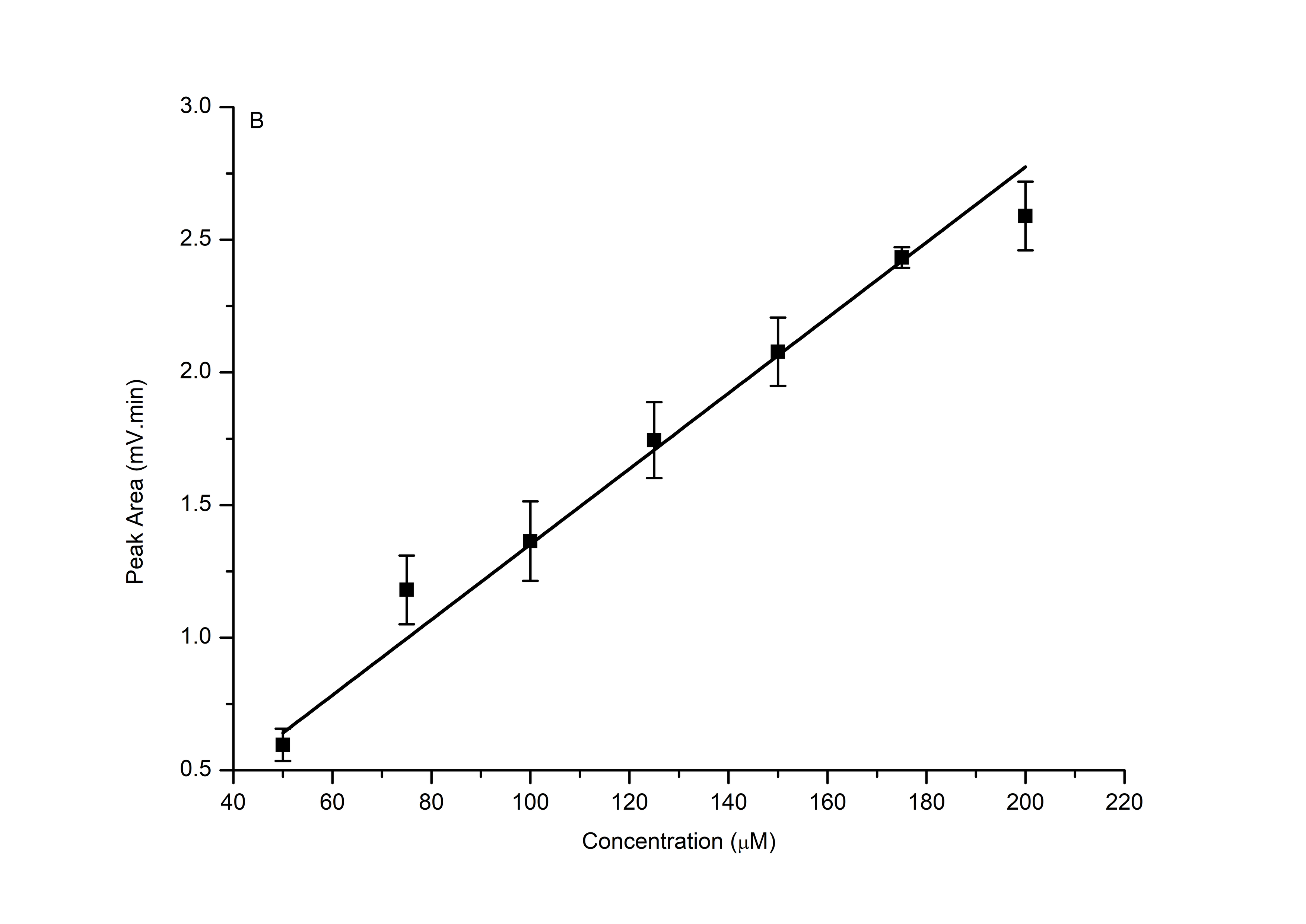


**Fig. S11** (A) Calibration curve for BA in CE-C^4^D with range 50-200 µM. Conditions: capillary temperature, 25 °C; voltage, + 20 kV; BGE, 30 mM borate buffer; buffer pH 9.2. Injection: 70 mbar for 10 seconds. C^4^D conditions: 750 kHz, Full scale 0.05 V, Amplitude 100%, Filter 1 Hz. (B) Linearity of the C^4^D detector responses for range 50-200 µM, R^2^=0,992.

The calibration curve for benzoic acid was constructed using seven concentration levels in the range of 50-200 µM. The regression equation was obtained with a correlation coefficient (R²) of 0.992, demonstrating good linearity.

The electropherograms presented for de calibration curves in DAD and C^4^D detectors show slight shifts in absolute migration times between different concentration levels. These variations are primarily attributed to the lack of an integrated cooling system in the external C^4^D and DAD detection cells, making the electroosmotic flow (EOF) sensitive to minor fluctuations in ambient temperature across different experimental days. However, it is important to emphasize that peak resolution and selectivity remained unaffected, and quantification was accurately performed using the corrected peak area to compensate for these velocity variations, as per standard capillary electrophoresis practices.


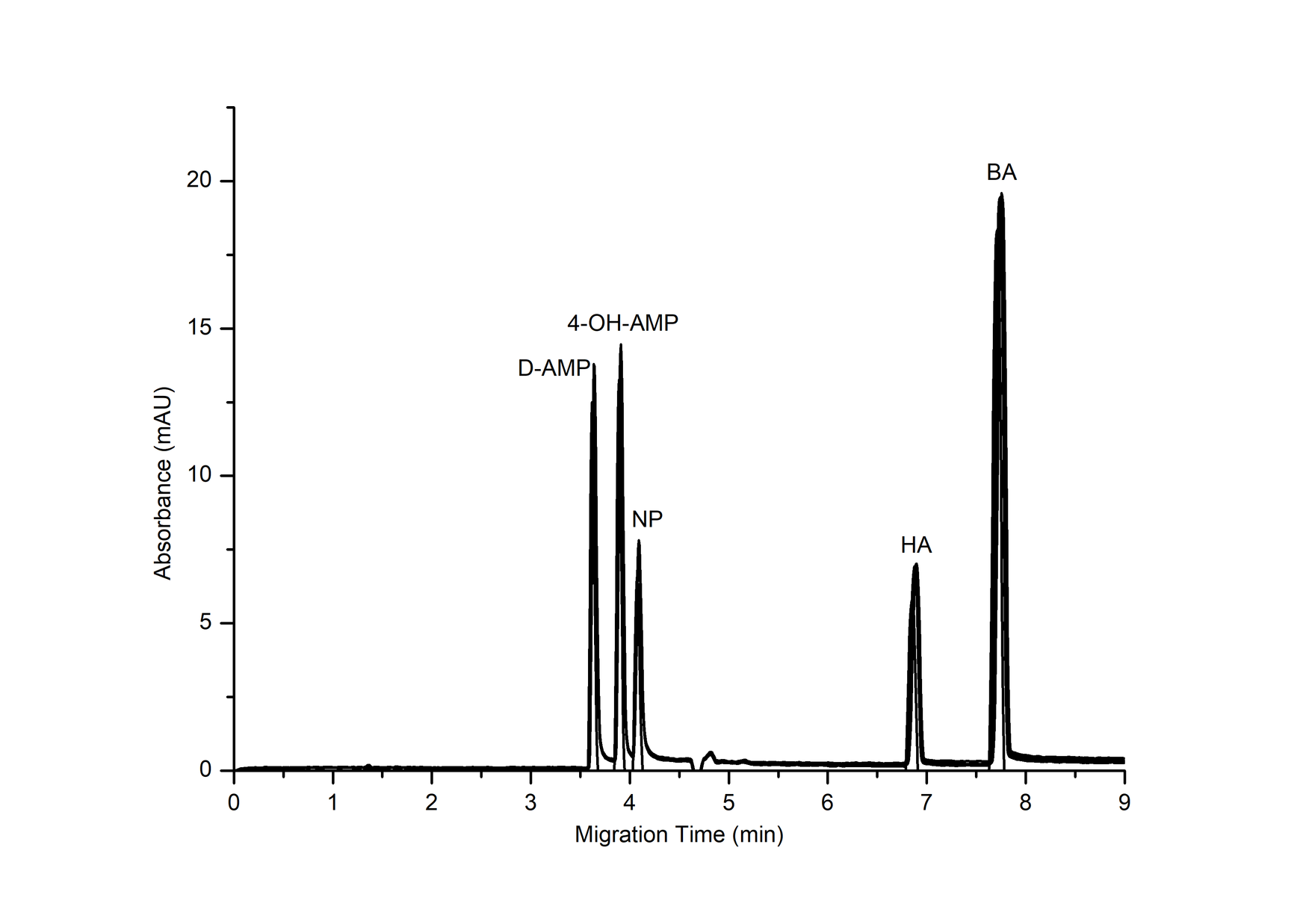


**Fig. S12** Electropherograms overlay of the intra-day repeatability test (n=6) using CE-DAD for dextroamphetamine (D-AMP) sulfate, 4-hydroxyamphetamine (4-OH-AMP), norephedrine hydrochloride (NP), hippuric acid (HA), and benzoic acid (BA). Conditions: Analyte concentrations: 100 µM in BGE containing 10% synthetic urine; Background Electrolyte (BGE): 30 mM borate buffer (pH 9.2); Capillary temperature: 25 °C; Voltage: +20 kV; Injection: 70 mbar for 10 s; UV Detection: 195 nm. C^4^D parameters (for simultaneous detection): 750 kHz, Amplitude 100%, Filter 1 Hz.

The intra-day repeatability was evaluated by performing six replicates (n=6) of the same sample under identical conditions within the same day. The relative standard deviations (RSDs) obtained for peak areas were below 2.5% for DAD, confirming the excellent repeatability of the method.

##
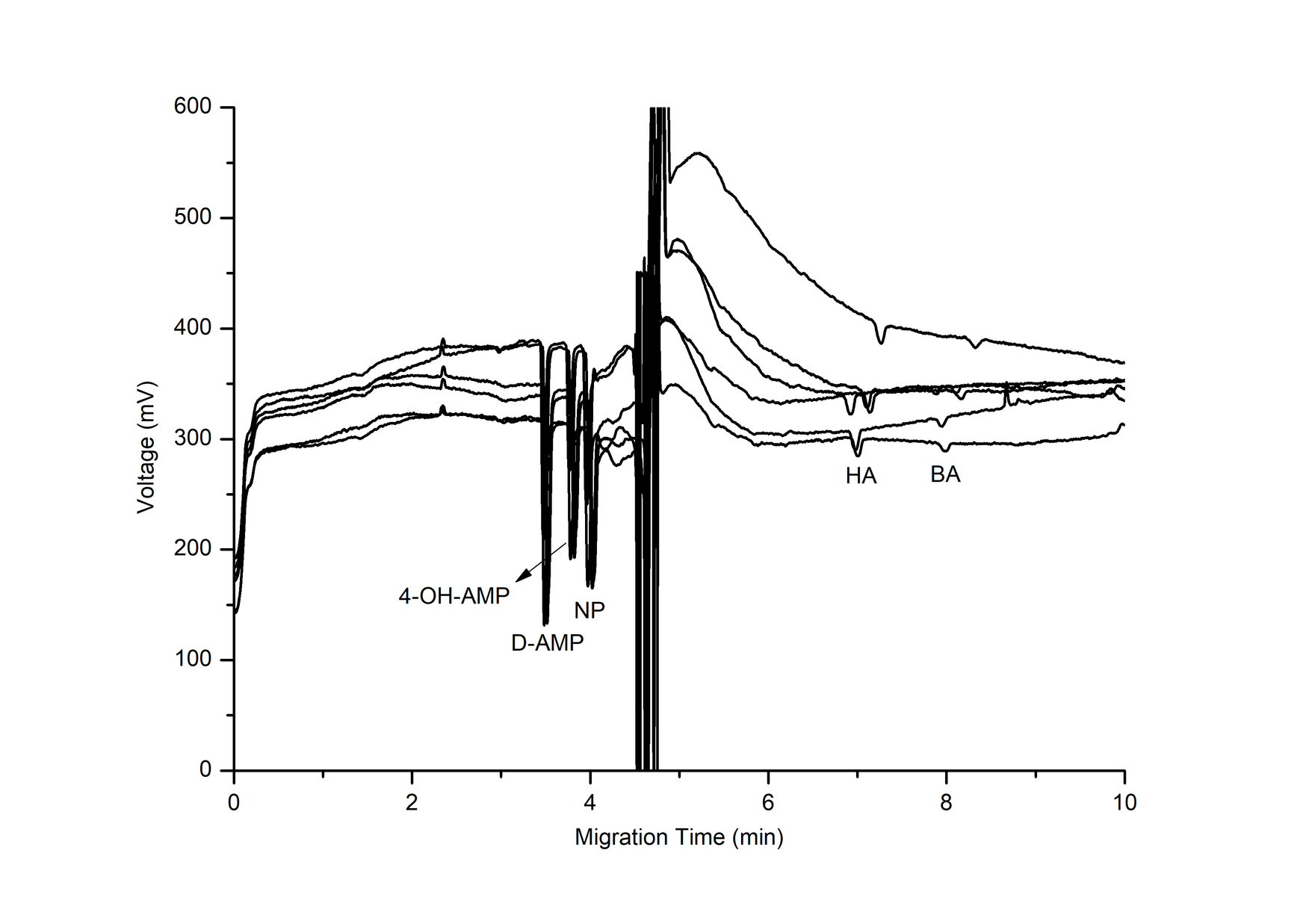


**Fig. S13** Electropherograms overlay of the intra-day repeatability test (n=6) using CE-C^4^D for dextroamphetamine (D-AMP) sulfate, 4-hydroxyamphetamine (4-OH-AMP), norephedrine hydrochloride (NP), hippuric acid (HA), and benzoic acid (BA). Conditions: Analyte concentrations: 100 µM in BGE containing 10% synthetic urine; Background Electrolyte (BGE): 30 mM borate buffer (pH 9.2); Capillary temperature: 25 °C; Voltage: +20 kV; Injection: 70 mbar for 10 s; UV Detection: 195 nm. C^4^D parameters (for simultaneous detection): 750 kHz, Amplitude 100%, Filter 1 Hz.

The intra-day repeatability was evaluated using six replicate measurements of the same sample under identical conditions within the same day, while intermediate precision was assessed over two separate days (n=11). The Relative Standard Deviations (RSDs) obtained for the C^4^D were higher than those observed for the DAD detector. While DAD precision was consistently high (RSD < 2.3%), the C^4^D detector showed higher variability, ranging from 1.56% to 16.42% for intra-day repeatability and from 5.21% to 25.80% for intermediate precision, particularly for benzoic acid. This behavior is attributed to the high sensitivity of the C^4^D to temperature fluctuations in the modular system. The high stability of the system is visually demonstrated in the overlay of six electropherograms provided in Figures S12 and S13.

**Table S1.** Recovery Test in human urine for CE-DAD-C^4^D

|  | | **DAD** | | | **C^4^D** | | |
| --- | --- | --- | --- | --- | --- | --- | --- |
| **Analyte** | **RSD (%)**  **25 µM** | | **RSD (%)**  **50 µM** | **RSD (%)**  **75 µM** | **RSD (%)**  **25 µM** | **RSD (%)**  **50 µM** | **RSD (%)**  **75 µM** |
| D-AMP | 21.7% | | 45.2% | 24.2% | 23.7% | 7.3% | 5.8 |
| 4-OH-AMP | 19.3% | | 12.4 | 4.1% | 36.2% | 3.3% | 8.2 |
| NP | 30.3% | | 19.7 | 3.1 | 6.2% | 32.6% | 5.7 |
| HA | 29.9% | | 1.8 | 5.1% | 10.7% | 5.4% | 5.4 |
| BA | 17.4% | | 1.8 | 7.6 | 38.4 | 1.2% | 9.8 |

The results presented in Table S1 illustrate the precision of the CE-DAD-C^4^D system when handling a complex biological matrix (raw human urine) without prior cleanup. The higher RSD% values observed for some analytes, particularly at lower concentrations (25 µM) and for D-AMP in the DAD detector (45.2% at 50 µM), are directly attributed to the presence of endogenous urinary chromophores and baseline fluctuations inherent to the 'dilute-and-shoot' approach. These experimental findings provide a clear justification for the implementation of the SPE procedure described in the main manuscript, which is essential to achieve the high precision (RSD < 5%) required for analytical applications. Notably, the complementarity of the dual-detection system is demonstrated by the C^4^D detector’s ability to provide reliable results (e.g., RSD of 7.3% and 3.3% for D-AMP and 4-OH-AMP at 50 µM, respectively) even when the DAD signal was compromised by matrix interferences.

**
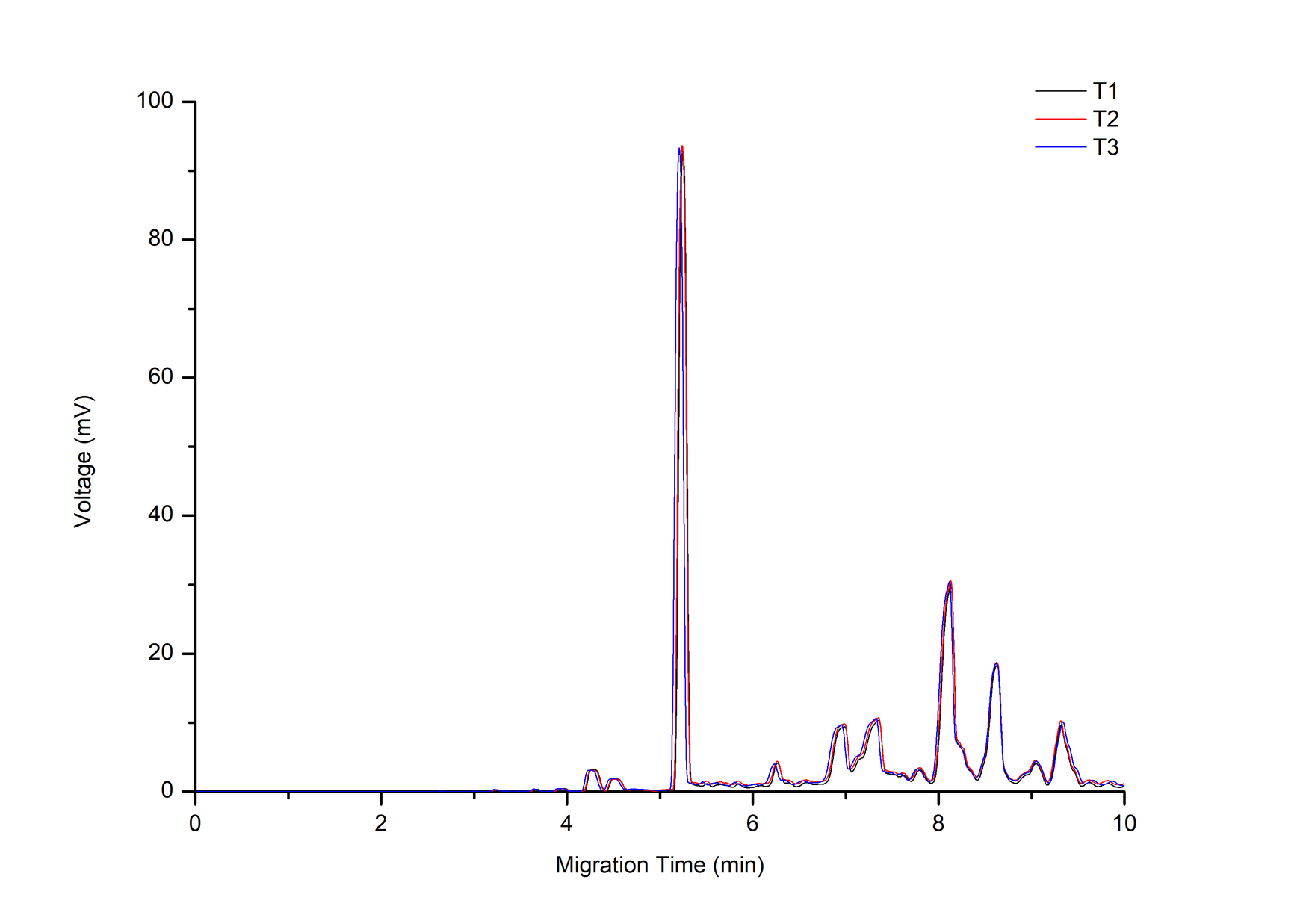
**

**Figure S14** Electropherograms overlay of the intra-day repeatability test in human urine (n=3) using CE-DAD for dextroamphetamine (D-AMP) sulfate, 4-hydroxyamphetamine (4-OH-AMP), norephedrine hydrochloride (NP), hippuric acid (HA), and benzoic acid (BA). Conditions: Analyte concentrations: 50 µM in BGE containing 10% synthetic urine; Background Electrolyte (BGE): 30 mM borate buffer (pH 9.2); Capillary temperature: 25 °C; Voltage: +20 kV; Injection: 70 mbar for 10 s; UV Detection: 195 nm. C^4^D parameters (for simultaneous detection): 750 kHz, Amplitude 100%, Filter 1 Hz. Samples were prepared by a simplified protocol consisting of centrifugation in a benchtop microcentrifuge for 10 min at maximum speed, followed by filtration through 0.22 µm membranes and 1:10 dilution in BGE.

The overlay of three independent injections demonstrates high instrumental stability and migration time repeatability, even when analyzing a complex, non-purified matrix. The visible baseline fluctuations and background signal profile under the 'dilute-and-shoot' conditions highlight the matrix effects from endogenous urinary compounds, visually supporting the necessity of the SPE cleanup procedure detailed in the main manuscript to achieve optimal analytical precision.


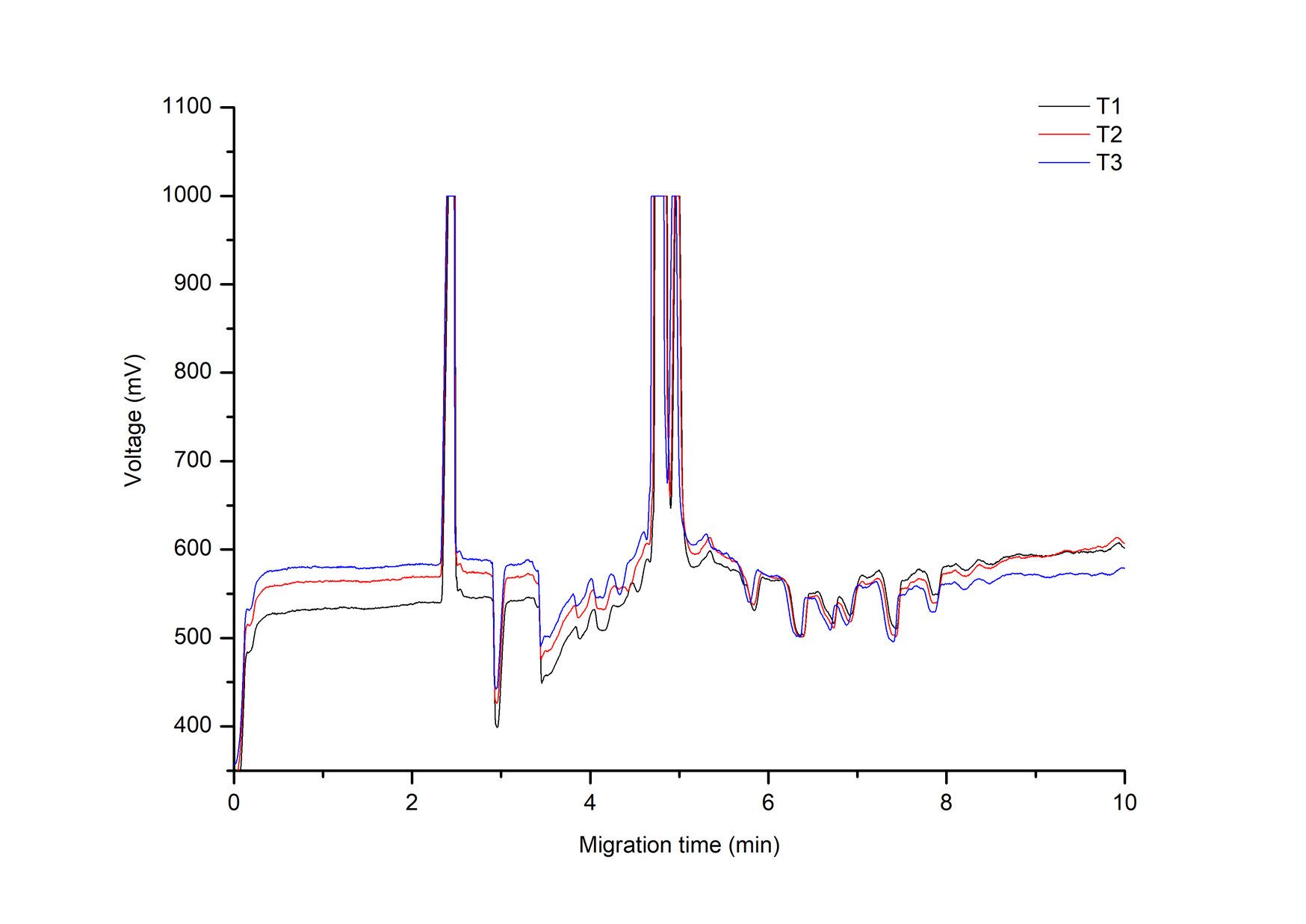


**Figure S15** Electropherograms overlay of the intra-day repeatability test in human urine (n=3) using CE-C^4^D for dextroamphetamine (D-AMP) sulfate, 4-hydroxyamphetamine (4-OH-AMP), norephedrine hydrochloride (NP), hippuric acid (HA), and benzoic acid (BA). Conditions: Analyte concentrations: 50 µM in BGE containing 10% synthetic urine; Background Electrolyte (BGE): 30 mM borate buffer (pH 9.2); Capillary temperature: 25 °C; Voltage: +20 kV; Injection: 70 mbar for 10 s; UV Detection: 195 nm. C^4^D parameters (for simultaneous detection): 750 kHz, Amplitude 100%, Filter 1 Hz. Samples were prepared by a simplified protocol consisting of centrifugation in a benchtop microcentrifuge for 10 min at maximum speed, followed by filtration through 0.22 µm membranes and 1:10 dilution in BGE.

Comparative Performance of DAD and C^4^D Detectors in Raw Urine: The complementary nature of the dual-detection system is illustrated by comparing the electropherograms in Figures S14 and S15. In the C^4^D profiles, a significantly cleaner baseline and well-defined peaks are observed compared to the DAD traces. This is because conductivity-based detection is inherently selective toward ionic species and blind to the vast majority of co-eluting organic urinary chromophores that disrupt the UV baseline. Conversely, the DAD signal shows prominent matrix interferences within the migration window of the target analytes. This clear disparity highlights the strength of orthogonal dual-detection: while C^4^D mitigates matrix-induced baseline instability for complex biological samples, the combined data provides a comprehensive analytical profile, visually supporting the implementation of the SPE cleanup for high-accuracy clinical applications.


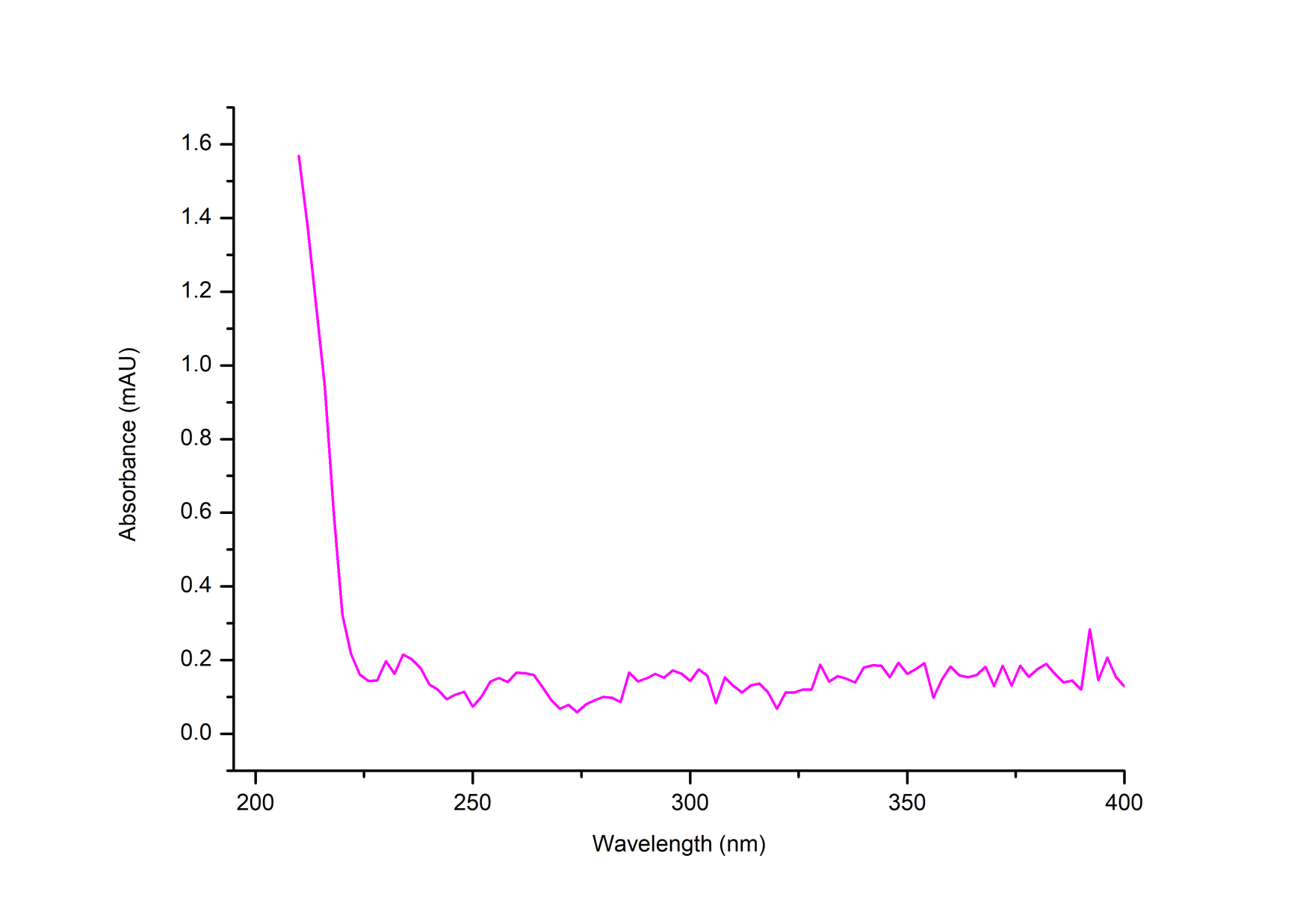


**Fig. S16** UV absorbance spectrum of Dextroamphetamine sulfate. Peak IDs/analyte concentrations: 100 µM. Conditions: capillary temperature, 25 °C; voltage, + 20 kV; BGE, 30 mM borate buffer; buffer pH 9.2. Injection: 70 mbar for 10 seconds.

The UV-Vis absorption spectrum for dextroamphetamine sulfate (Figure S16) is characteristic of a phenylethylamine with an unsubstituted benzene ring, showing strong absorption in deep UV (before 200 nm). The absorbance rapidly declines to close to zero at longer wavelengths. Like norephedrine, dextroamphetamine is a weak chromophore in the 230-300 region, making detection at short wavelengths imperative to maximize the signal. This spectral profile, together with the other analytes, justifies the selection of the 195 nm wavelength for the construction of the calibration curves and calculation of the detection and quantification limits, however the other wavelengths continued to be monitored in the method, ensuring the necessary sensitivity for all the compounds under study in this work.

**
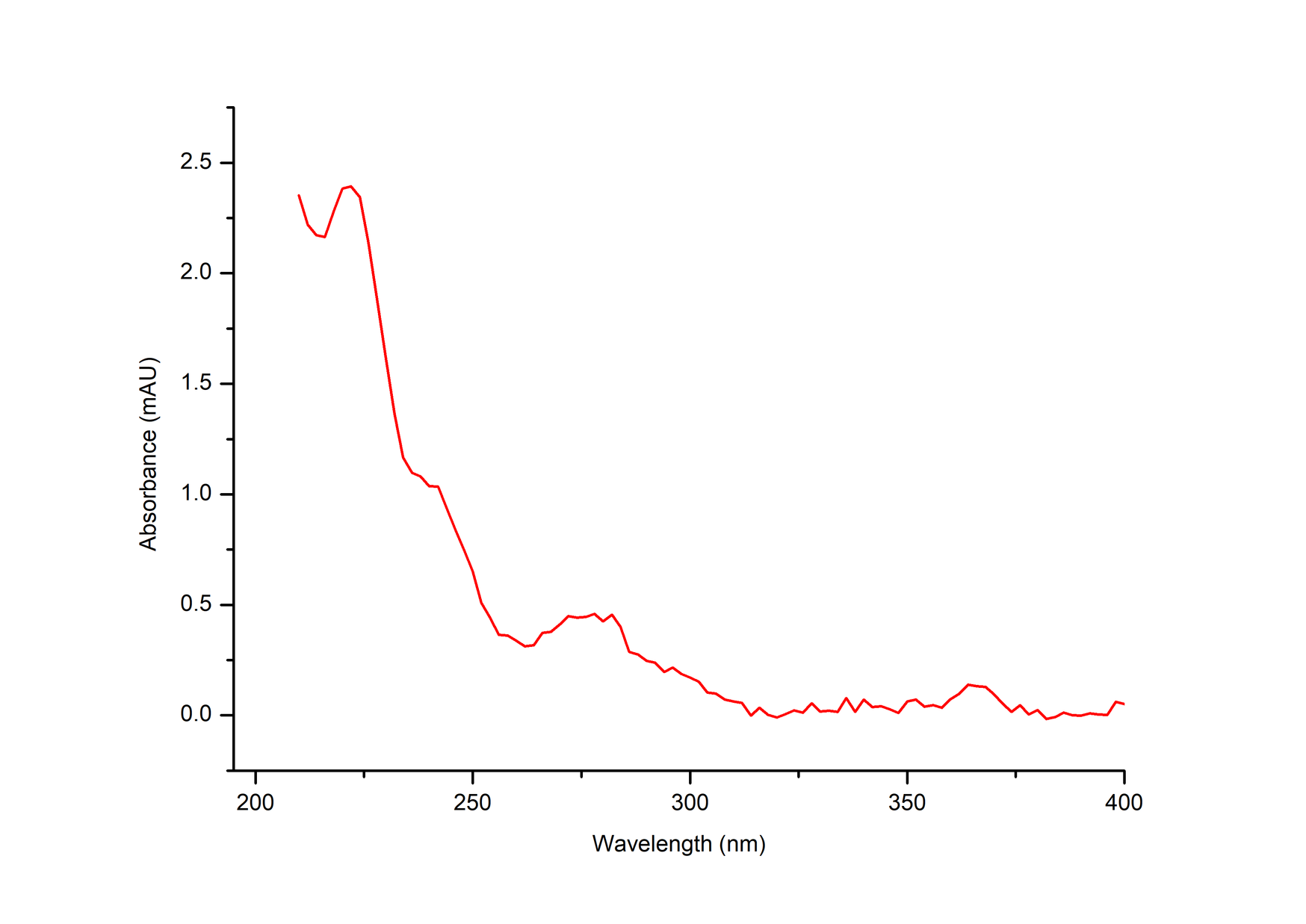
**

**Fig. S17** UV absorbance spectrum of 4-hydroxyamphetamine. Peak IDs/analyte concentrations: 100 µM. Conditions: capillary temperature, 25 °C; voltage, + 20 kV; BGE, 30 mM borate buffer; buffer pH 9.2. Injection: 70 mbar for 10 seconds.

Based on the UV-Vis absorption spectrum of 4-hydroxyamphetamine (Figure S17), which is representative of the chromophoric nature of the analytes, a peak of high absorbance was observed at 205-210 nm. Aiming at the maximum sensitivity of the method, the wavelength of 195 nm was selected for quantification, as it provided the highest signal response for all analytes tested. Although calibration curves and primary results are presented and calculated at 195 nm for standardization and sensitivity optimization, the Capillary Electrophoresis (CE) method coupled to the Diode Array Detector (DAD) continued to simultaneously monitor wavelengths of 210, 220, 230, 250, 260, 265, and 270 nm. This multi-wavelength monitoring was maintained for spectral confirmation of the purity and identity of the peaks, exploiting the secondary absorption bands to increase the robustness and selectivity of the method.

**
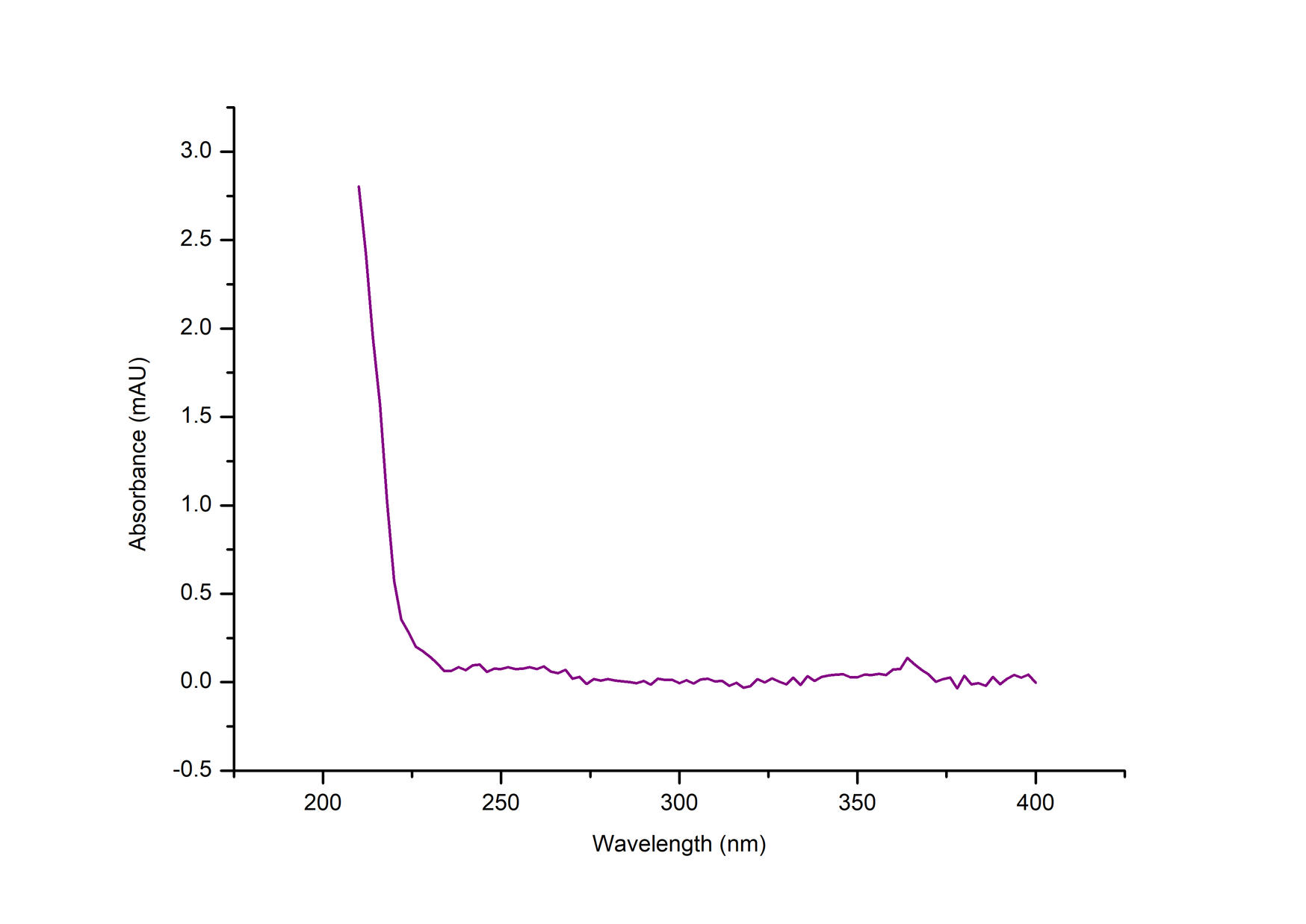
**

**Fig. S18** UV absorbance spectrum of norephedrine hydrochloride. Peak IDs/analyte concentrations: 100 µM. Conditions: capillary temperature, 25 °C; voltage, + 20 kV; BGE, 30 mM borate buffer; buffer pH 9.2. Injection: 70 mbar for 10 seconds.

The UV-Vis absorption spectrum for norephedrine hydrochloride (Figure S18) reveals the absorption profile typical of an unsubstituted aromatic ring, characterized by a high-energy transition band. Maximum absorption is observed at wavelengths below 200 nm, with absorbance rapidly dropping to baseline (near zero) after 230 nm. The absence of significant absorption bands at longer wavelengths (> 230 nm) confirms the dependence of norephedrine on deep UV detection. This spectral profile justifies the choice of the 195 nm wavelength for quantification, to capitalize on the maximum sensitivity available for this analysis.

**
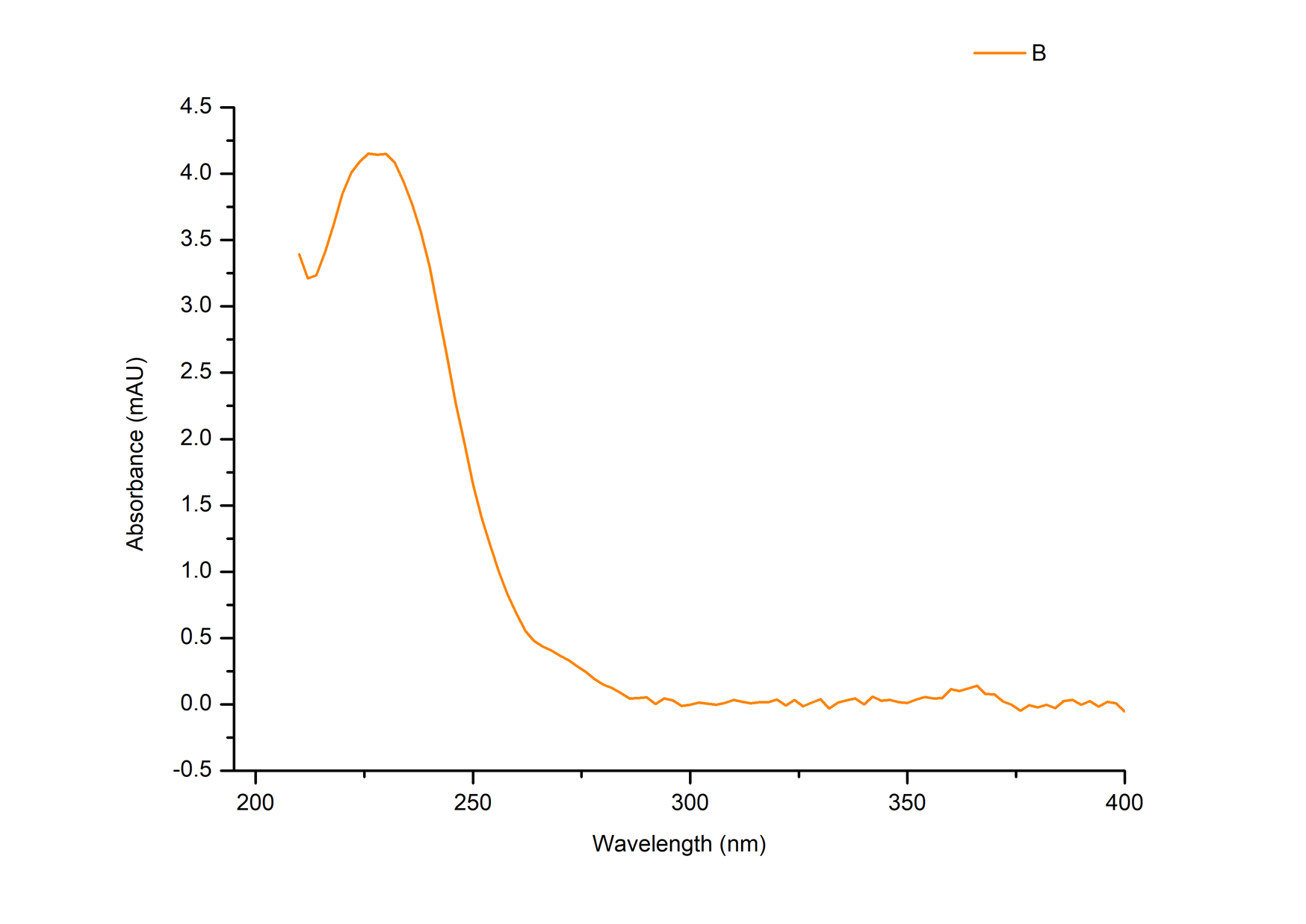
**

**Fig. S19** UV absorbance spectrum of hippuric acid. Peak IDs/analyte concentrations: 100 µM. Conditions: capillary temperature, 25 °C; voltage, + 20 kV; BGE, 30 mM borate buffer; buffer pH 9.2. Injection: 70 mbar for 10 seconds.

The UV-Vis absorption spectrum for hippuric acid (Figure S19) reflects the strong absorption of the benzene aromatic ring and the carbonyl amide group. This chromophore results in a local absorption maximum (benzenoid B-band) at approximately 225 nm. However, the absolute maximum absorptivity occurs in the deep UV region (E2-band, < 200 nm). Absorbance rapidly declines to baseline after 300 nm, with no significant secondary bands. This spectral profile confirms that hippuric acid is a strong chromophore, with its highest signal response occurring in the far-UV region, which supports the choice of 195 nm for the analyte panel-wide quantification to maximize overall method sensitivity.


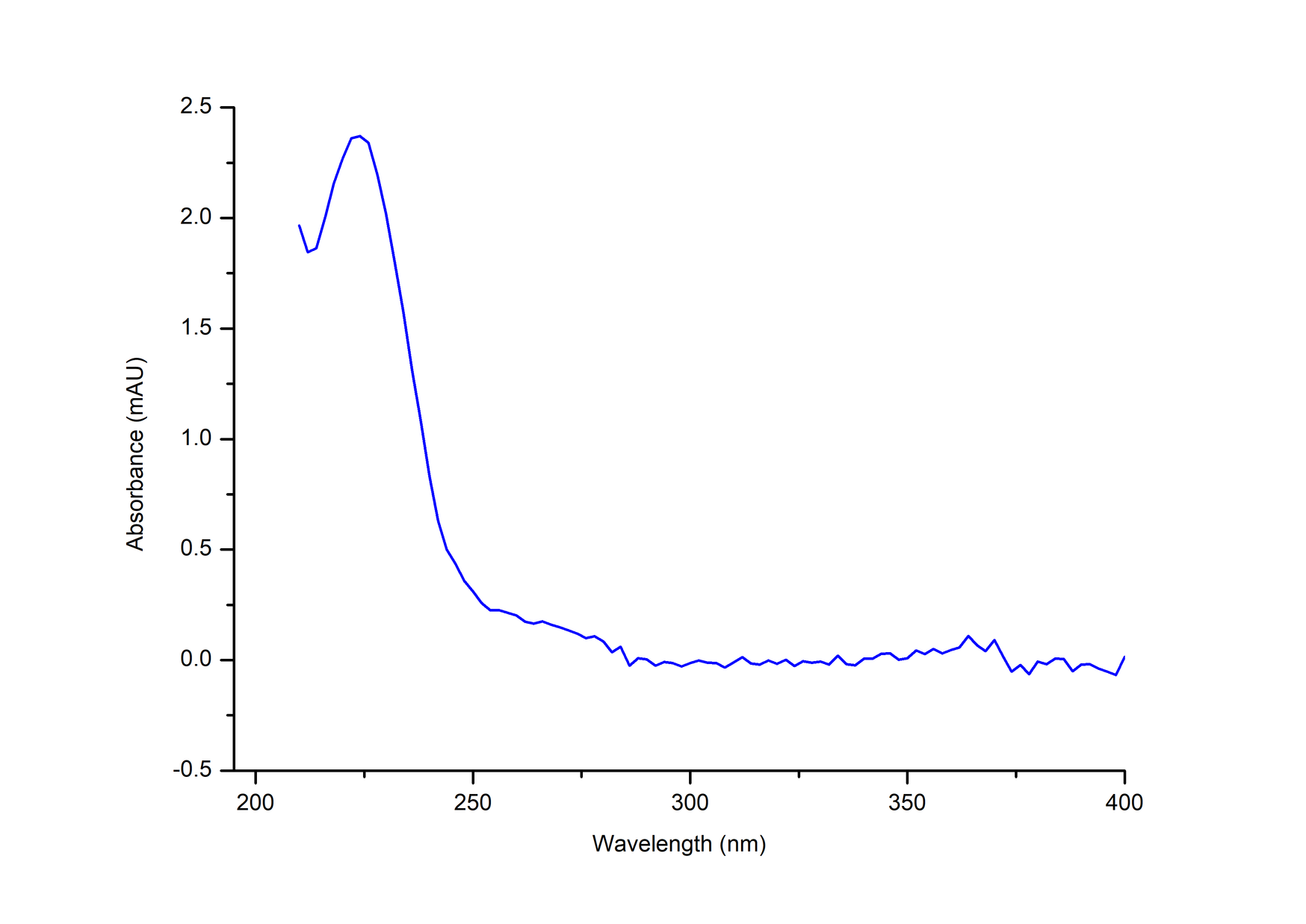


**Fig. S20** UV absorbance spectrum of benzoic acid. Peak IDs/analyte concentrations: 100 µM. Conditions: capillary temperature, 25 °C; voltage, + 20 kV; BGE, 30 mM borate buffer; buffer pH 9.2. Injection: 70 mbar for 10

The UV-Vis absorption spectrum for benzoic acid (Figure S20) exhibits a high-intensity profile characteristic of a conjugated π system. A significant local absorption peak occurs at approximately 225 nm, similar to hippuric acid. The presence of the carboxyl group conjugated to the benzene ring is responsible for this strong band, with secondary absorption extending as a weak shoulder between 270–280 nm. Although these secondary bands are present, the molar absorptivity in the deep UV region (E2-band at 195 nm) is substantially higher for all chromophoric analytes. Consequently, 195 nm was selected for the CE-DAD method to ensure maximum analytical sensitivity for the complete panel of compounds, especially for those with negligible absorption at higher wavelengths.


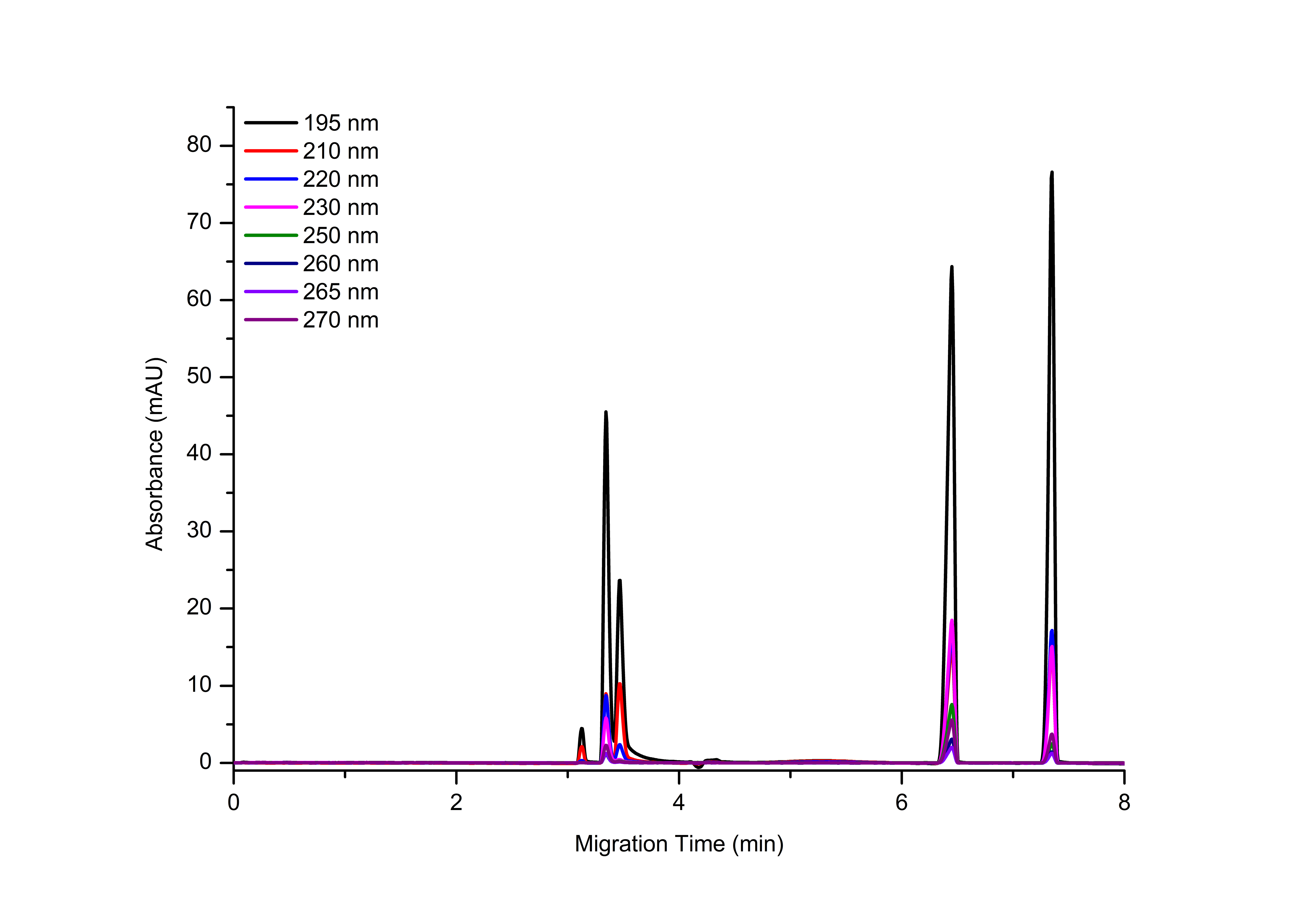


**Fig. S21** Overlaid electropherograms of a standard mixture containing D-AMP and its metabolites monitored at multiple wavelengths (195–270 nm). Peak IDs/analyte concentrations: 50 µM. Conditions: capillary temperature, 25 °C; voltage, + 20 kV; BGE, 30 mM borate buffer; buffer pH 9.2. Injection: 70 mbar for 10 s. The highest analytical signal was achieved at 195 nm.

To ensure maximum sensitivity for the simultaneous determination of D-amphetamine (D-AMP) and its major metabolites (4-OH-AMP, NP, HA, and BA), a multi-wavelength screening was performed using the Diode Array Detector (DAD). Figure S21 illustrates the overlaid electropherograms monitored from 195 nm to 270 nm. As observed, 195 nm provided the highest analytical signal (peak height and area) for all analytes. This is particularly critical for D-AMP, which exhibits a strong absorption band in the far-UV region (π-π* transition, E2-band of the aromatic ring) but shows a sharp decrease in molar absorptivity at wavelengths above 210 nm. Although metabolites such as hippuric acid (HA) and benzoic acid (BA) show local absorption maxima at higher wavelengths (e.g., the benzenoid B-band at 225 nm), the signal-to-noise ratio (S/N) at 195 nm remains superior for the entire mixture due to higher molar absorptivity in the deep UV region. Therefore, 195 nm was selected as the optimal analytical compromise to achieve the lowest limits of detection (LOD) and quantification (LOQ) in a single analytical run. The method was validated at this wavelength (195 nm), confirming that no significant interference from the background electrolyte (BGE) or the urine matrix affects the detection of the analytes.

**
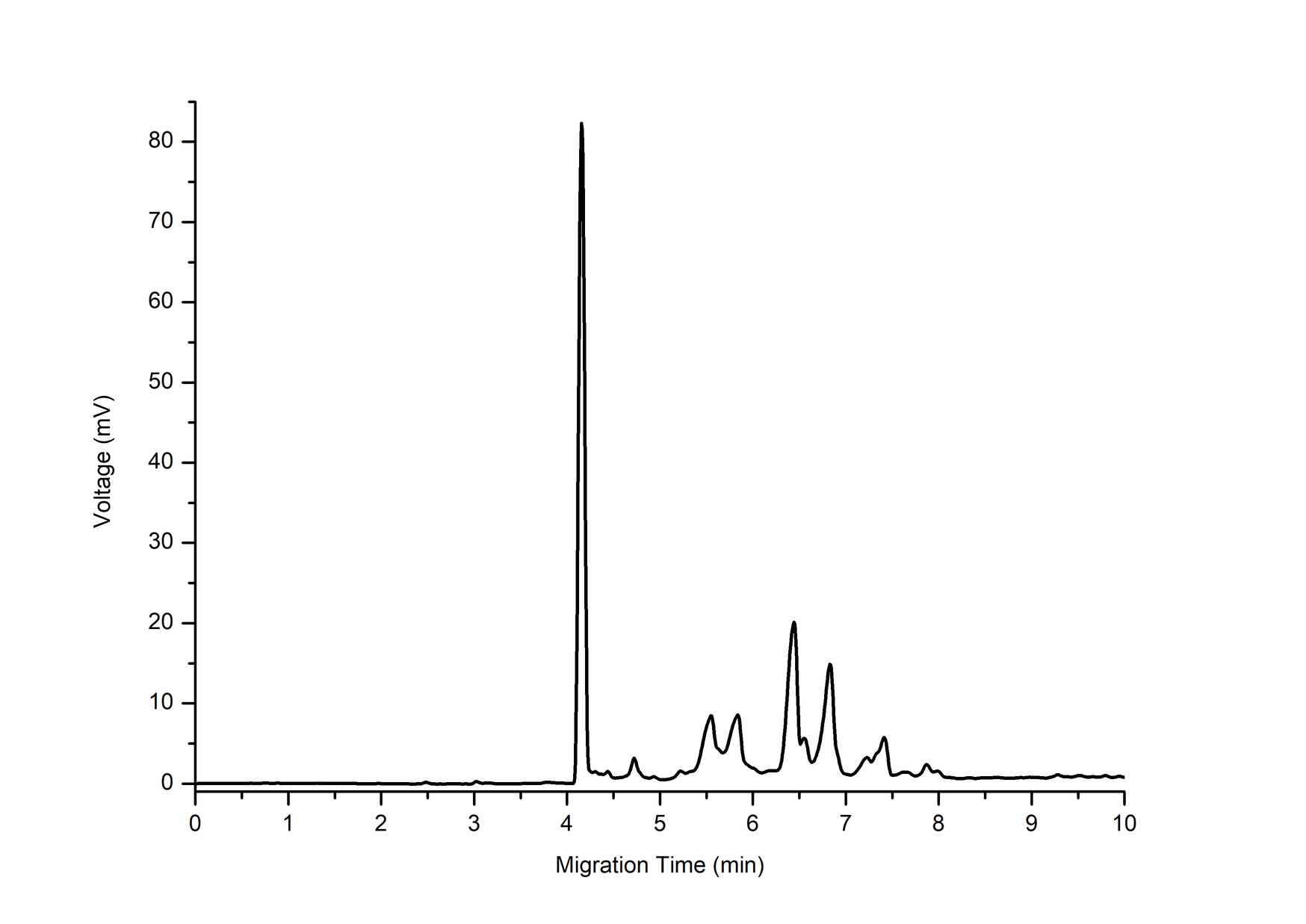
Fig. S22.** Electropherogram of a blank for human urine sample in the background electrolyte in CE-DAD. Conditions: capillary temperature, 25 °C; voltage, + 20 kV; BGE, 30 mM borate buffer; buffer pH 9.2. Injection: 70 mbar for 10 seconds. UV: 195 nm. Samples were prepared by a simplified protocol consisting of centrifugation in a benchtop microcentrifuge for 10 min at maximum speed, followed by filtration through 0.22 µm membranes and 1:10 dilution in BGE.

The selectivity of the developed CE-DAD-C^4^D method was rigorously evaluated by analyzing blank urine samples from healthy volunteers (diluted 1:10 v/v in BGE). As demonstrated in Figure S22, the electropherogram obtained at 195 nm shows a stable baseline with no endogenous compounds co-eluting at the migration times of d-amphetamine (approx. 4.5 min) and its metabolites. The prominent peak observed at approximately 4.1 minutes corresponds to the electroosmotic flow (EOF). The minor signals appearing after 5 minutes are attributed to common urinary electrolytes and do not interfere with the quantification of the target analytes. This profile confirms that the sample preparation (simple dilution) is sufficient to eliminate potential matrix interferences, ensuring the accuracy of the results for clinical applications."

**
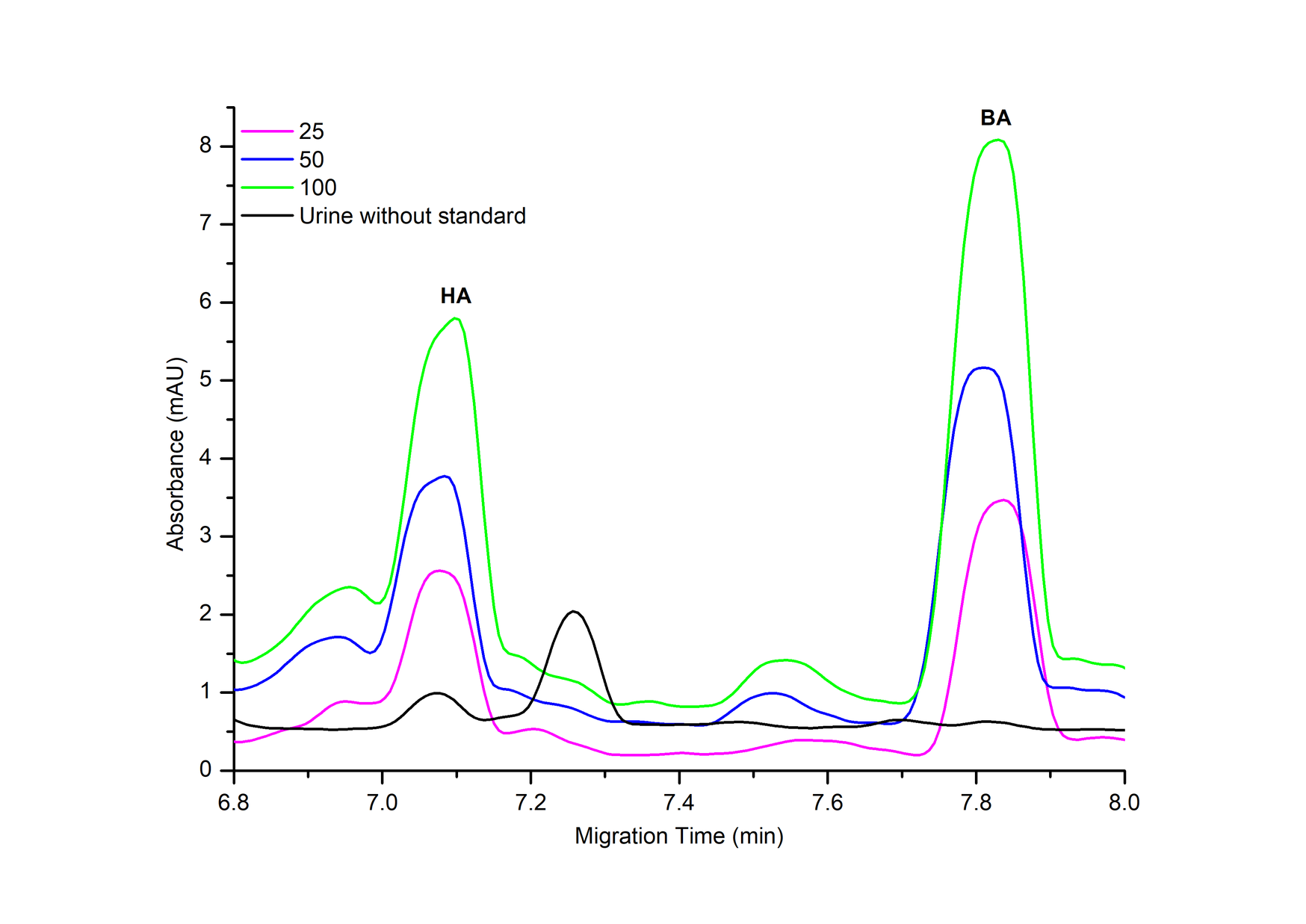
**

**Fig. S23** Test for quantification of hippuric acid and benzoic acid in human urine sample mix using the method Standard Addition Method with 25, 50, 100 µM of Hippuric Acid and Benzoic Acid. Conditions: capillary temperature, 25 °C; voltage, + 20 kV; BGE, 30 mM borate buffer; buffer pH 9.2. Injection: 70 mbar for 10 seconds. UV: 195 nm.

For the quantification of Hippuric Acid (HA) and Benzoic Acid (BA) in a commercially acquired human urine sample, the standard addition method was employed, by spiking the sample with three distinct standard concentrations (25, 50 and 100 µM). The analysis detected endogenous peaks for HA and BA. The linearity of the analytical curves was excellent, with high coefficients of determination R^2^=0.9922 for HA and R^2^= 0.9919 for BA, validating the precision of the regression model. Based on this linear regression, the basal concentrations in the urine sample were calculated. These results demonstrate the robustness and applicability of the Capillary Electrophoresis (CE) method for the reliable quantification of these metabolites in complex biological matrices.
